# Current Status of Novel Agents for the Treatment of B Cell Malignancies: What’s Coming Next?

**DOI:** 10.3390/cancers14246026

**Published:** 2022-12-07

**Authors:** Mariana Tannoury, Delphine Garnier, Santos A. Susin, Brigitte Bauvois

**Affiliations:** Centre de Recherche des Cordeliers, Sorbonne Université, Université Paris Cité, Inserm, Cell Death and Drug Resistance in Lymphoproliferative Disorders Team, F-75006 Paris, France

**Keywords:** leukemia, lymphoma, biomarker, inhibitor, signaling, survival, drug resistance, antibody, therapy, metabolism

## Abstract

**Simple Summary:**

Since the identification of a large variety of biomarkers in B cell malignancies as being driving factors for tumor progression and patient prognosis, their targeting may confer valuable options for the treatment of these diseases. Over the past 20 years, the permanent development of a multitude of inhibitors acting on survival-associated biomarkers has made it into clinical evaluation in B lymphoid tumors. Although certain drugs approved by the US Food and Drug Administration improve clinical outcome, some patients do not respond and others relapse. This review summarizes the current state-of-the-art, provides a summary of new, safer, more selective inhibitors currently under evaluation in clinical trials, and highlights the emerging positioning of metabolic drugs in tumor B cell biology as a promising strategy to be translated into clinical practice.

**Abstract:**

Resistance to death is one of the hallmarks of human B cell malignancies and often contributes to the lack of a lasting response to today’s commonly used treatments. Drug discovery approaches designed to activate the death machinery have generated a large number of inhibitors of anti-apoptotic proteins from the B-cell lymphoma/leukemia 2 family and the B-cell receptor (BCR) signaling pathway. Orally administered small-molecule inhibitors of Bcl-2 protein and BCR partners (e.g., Bruton’s tyrosine kinase and phosphatidylinositol-3 kinase) have already been included (as monotherapies or combination therapies) in the standard of care for selected B cell malignancies. Agonistic monoclonal antibodies and their derivatives (antibody–drug conjugates, antibody–radioisotope conjugates, bispecific T cell engagers, and chimeric antigen receptor-modified T cells) targeting tumor-associated antigens (TAAs, such as CD19, CD20, CD22, and CD38) are indicated for treatment (as monotherapies or combination therapies) of patients with B cell tumors. However, given that some patients are either refractory to current therapies or relapse after treatment, novel therapeutic strategies are needed. Here, we review current strategies for managing B cell malignancies, with a focus on the ongoing clinical development of more effective, selective drugs targeting these molecules, as well as other TAAs and signaling proteins. The observed impact of metabolic reprogramming on B cell pathophysiology highlights the promise of targeting metabolic checkpoints in the treatment of these disorders.

## 1. Background and Introduction

**Figure 1:**
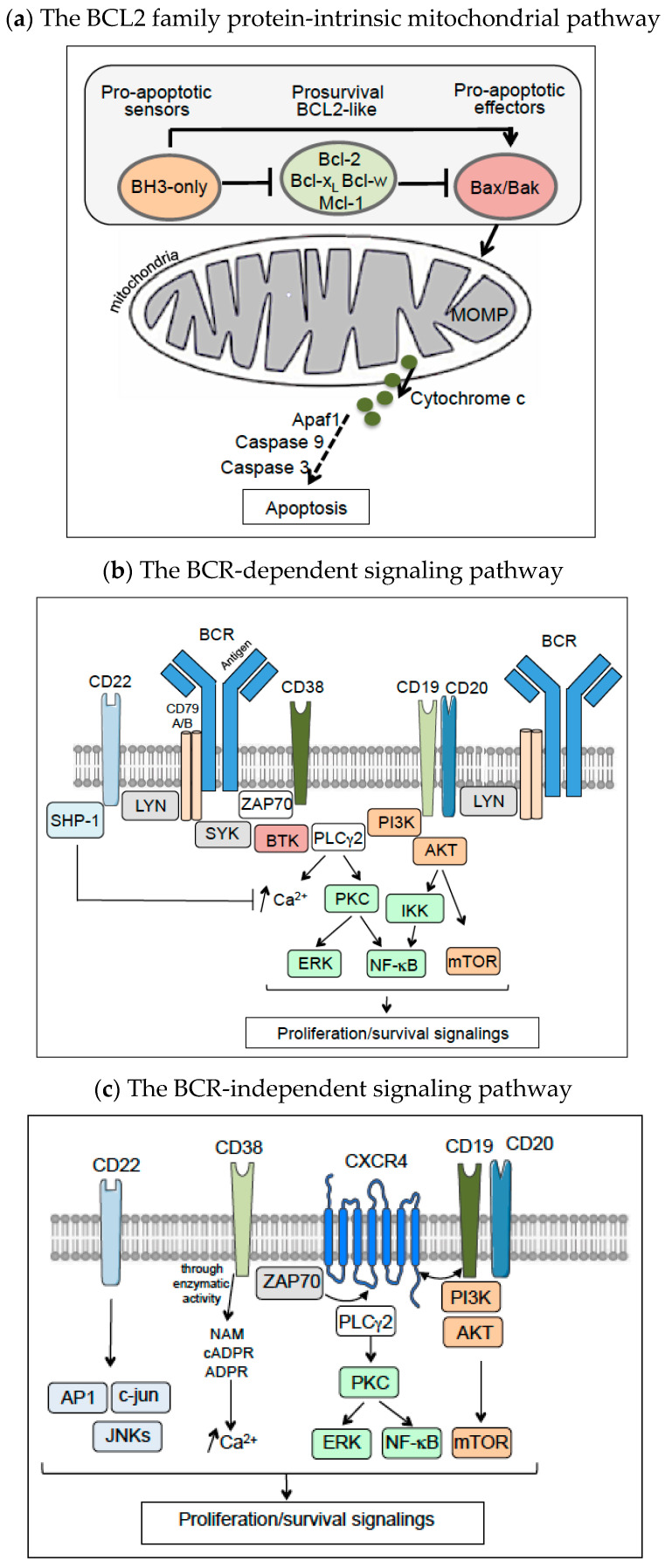
Models for the signaling pathways regulated by BCL2 family proteins, BCR components, and TAAs (CD19, CD20, CD22, CD38) in B cell malignancies. (**a**) Prosurvival BCL2-like proteins (Bcl-2, Bcl-x_L_, Bcl-w, and Mcl-1) interact with the proapoptotic members called BH3-only proteins (Bim, Bid, Puma, Bad, and Noxa), or bind to the apoptotic effectors Bax and Bak and sequester them in an inactive form. The pro-apoptotic proteins induce apoptosis by activating Bax and Bak either directly or indirectly through inhibition of the anti-apoptotic proteins. Once activated, Bax and Bak form oligomers; this leads to pore formation, mitochondrial outer membrane permeabilization (MOMP), and then the release of cytochrome c and other pro-death proteins from mitochondria, resulting in caspase activation and cell apoptosis. (**b**) Upon BCR ligation and activation, Lyn phosphorylates CD79A/B, which activates SYK. In turn, activated SYK phosphorylates and recruits the B-cell linker protein (BLNK), which binds to BTK and PLCγ2 and catalyzes the cleavage of membrane phosphatidyl inositol bisphosphate into inositol triphosphate and diacyl glycerol. This releases Ca^2+^ from intracellular stores and activates PKCβ and downstream proteins. The positive BCR coreceptors CD19 and CD20 are phosphorylated by LYN during BCR signaling, leading to the recruitment of PI3K to the BCR. Together, these signaling pathways activate the ERK, NF-κB, AKT/mTOR pathways. The negative B cell coreceptor CD22 is phosphorylated by LYN and inhibits the BCR signal by recruiting SHP-1, which dephosphorylates and inactivates BLNK and CD19 and thus leads to a decrease in the cytoplasmic Ca^2+^ concentration. SHP-1 also decreases the cytoplasmic Ca^2+^ concentration by activating the plasma membrane Ca^2+^-ATPase (PMCA4) and thus promoting Ca^2+^ efflux. CD38 activation leads to the phosphorylation of ZAP70 and further sustains the signal mediated by the BCR. (**c**) Independently of the BCR signaling pathway, CD19, CD20, CD22, and CD38 have a role in activating proliferation/survival pathways in malignant B cells. CD38 (via ZAP70 activation), CD19 and CD20 are linked to the proliferation/survival signaling pathways controlled by CXCR4. Through CD38′s enzymatic activity, the reaction products (nicotinamide adenine dinucleotide, cyclic ADP-ribose, and ADP-ribose) are used inside the cells to open different Ca^2+^ stores, which leads to an increase in the cytoplasmic Ca^2+^ concentration independently of the conventional IP3 pathway. CD22 ligation activates AP-1, c-jun, and the c-jun NH2-terminal kinases (JNKs).

The balance between cell death and cell proliferation contributes to the maintenance of tissue homeostasis. Cell death evasion is a hallmark of human cancers (including B cell malignancies; Table 1) and might contribute to treatment resistance [1]. A number of molecular pathways underpin cell growth and cell survival in B cell malignancies (Figure 1). For example, anti-apoptotic proteins from the B-cell lymphoma/leukemia 2 (BCL2) family (such as Mcl-1, Bcl-2, Bcl-x_L,_ and Bcl-w) are overexpressed in most B cell malignancies (Table 1) as a result of genetic lesions or changes in signal transduction [2,3]. This expression is associated with a poor prognosis [3,4,5,6]. Anti-apoptotic BCL2 proteins interact with the pro-apoptotic “BH3-only” proteins (such as Bim, Bid, Puma, Bad, and Noxa) or bind and sequester the apoptotic effectors Bax and Bak in an inactive form (Figure 1a) [3,7]. The pro-apoptotic proteins induce apoptosis by activating Bax and Bak either directly or indirectly via inhibition of the anti-apoptotic proteins (Figure 1a) [3,7]. Active B-cell receptor (BCR) signaling also contributes to cell survival, proliferation, and resistance to apoptosis in B cell malignancies [8,9,10,11,12] (Table 1). BCR-mediated proliferation of malignant B cells may be caused by BCR pathway mutations and/or chronic active stimulation of the BCR [8,9,10,11,12]. The mechanism of BCR pathway activation in B cell malignancies is complex and has been well reviewed by Burger and Wiestner [13]; it includes continuous BCR stimulation by microbial antigens or autoantigens, activating mutations within the BCR complex or downstream signaling partners, and antigen-independent BCR signaling [13]. The BCR pathway includes a large array of kinases, adaptor proteins and transcription factors (such as NF-κB); in particular, Bruton tyrosine kinase (BTK) and phosphatidylinositol-3 kinase (PI3K) are critical signaling enzymes involved in uncontrolled B cell proliferation and survival [10,11,12,13] (Figure 1b). Lastly, high levels of expression of certain tumor-associated antigens (TAAs; CD19, CD20, CD22, and CD38) in most B cell malignancies are correlated with a high proliferation rate and disease progression (Table 1) [14,15,16,17]. These TAAs are physically associated with the BCR [18,19,20,21] and are involved in modulating BCR-dependent (Figure 1b) and BCR-independent proliferation/survival signals (Figure 1c) [13,15,16,17,18,20,21,22,23,24,25,26].

Over the last 25 years, greater knowledge of the detrimental roles of these survival-associated proteins in malignant B cell pathologies have prompted the development of a large array of inhibitors. These novel anticancer agents include small molecules that target BCL2 members, BTK, or PI3K, and mAbs and their derivatives (mono/bispecific Ab, antibody–drug conjugate/ADC, antibody–radioisotope conjugate/ARC, bispecific T cell engager/BiTE, and chimeric antigen receptor-modified T cells/CAR-T) against TAAs. The use of some of these novel agents have been authorized by the US Food and Drug Administration (FDA) [27]. Although these drugs improve clinical outcomes when used alone or in combination with other treatments, some patients do not respond, and others relapse after an initial response [28,29,30,31,32,33,34]. As such, efforts to develop more effective, selective inhibitors against the same targets or new targets in malignant B cells must be continued. Specifically, novel mAbs (acting on other TAAs) and small-molecule inhibitors (acting on survival-associated proteins, such as signaling proteins, proteases, immune checkpoints, etc.) are in preclinical or clinical development. Here, we first review the current management of B lymphoid tumors with FDA-approved drugs targeting the above-cited TAAs, Bcl-2, or BTK/PI3K. We then focus on the clinical efficacy of newly developed drug candidates for inhibiting current and novel protein targets and overcoming some forms of resistance. Lastly, we review perspectives for therapy, with emphasis on the importance of metabolic reprogramming in tumor B cell biology and opportunities for inhibiting this process.

**Table 1:** Characteristics of the main B cell malignancies.

B Cell Malignancy	Pathophysiology	Expression of BCL2 Prosurvival Proteins	Active (Pre-)BCR Signaling	Surface Markers That Are Strongly Expressed	References
B-ALL	Transformation and expansion of lymphatic B progenitor cells	Bcl-2, Mcl-1, Bcl-x_L_	pre +	CD19 CD20 CD22 CXCR4	[7,35,36,37,38,39,40,41]
HCL	Accumulation of CD5^+^ B cells most in the blood, bone marrow and spleen	Bcl-2 > Mcl-1	+	CD19 CD20 CD22 CD38 CXCR4	[2,11,40,42,43]
CLL	Accumulation of CD5^+^ B cells primarily in the blood and bone marrow	High Bcl-2 > Mcl-1 >> Bcl-x_L_	+	CD19 CD20 CD38 (poor prognosis) BAFF-R ROR1 CXCR4	[3,7,36,38,40,41,44,45]
SLL (B-NHL)	Accumulation of CD5^+^ B cells most in the lymph nodes	Bcl-2, Mcl-1, Bcl-x_L_	+	CD19 CD20 CD38 (poor prognosis) ROR1	[40,44,45,46,47]
MM	Accumulation of clonal, Ig secreting plasma cells in the bone marrow	High Bcl-2, Mcl-1, Bcl-x_L_, Bcl-w	+	CD19 CD22 CD38 BCMA CD13 ADAM17 ROR1 CXCR4	[3,7,40,41,48,49,50,51]
FL (B-NHL)	Extensive proliferation and accumulation of abnormal B cells in lymph nodes	High Bcl-2, Bcl-w and Bcl-x_L_ > Mcl-1	−/+	CD19 CD20 CD22 CD38 CXCR4	[3,7,40,41,45,48,52]
MCL (B-NHL)	Development of abnormal B cells in the mantle zone of lymph nodes, spleen, bone marrow, blood, and gastrointestinal tract	High Bcl-w and Bcl-2 > Mcl-1	+	CD19 CD20 CD22 CD38 ROR1 CXCR4	[3,7,40,41,47,52,53]
MZL (B-NHL)	Development of abnormal B cells in the marginal zones of lymph tissue	High Bcl-2 and Bcl-w > Bcl-x_L_	+	CD19 CD20 CD22 CXCR4	[3,10,40,41,52,54,55]
DLBCL (B-NHL)	Development of abnormal B cells in germinal centers of secondary lymphoid organs	Bcl-2 and Bcl-w > Mcl-1, Bcl-x_L_	+	CD19 CD20 CD22 CD38 CXCR4	[3,7,36,40,41,45,52,56,57]
WM (B-NHL)	Proliferation of clonal, IgM-secreting plasma cells in the lymph nodes and bone marrow	High Bcl-2 > Bcl-x_L_ and Mcl-1	+	CD19 CD20 CD38 CXCR4 (mutation)	[8,40,46,58,59]

ADAM17, a disintegrin and metalloprotease 17; B-ALL, B-acute lymphoblastic leukemia; BAFF-R, B-cell activating factor receptor; BCMA, B cell maturation antigen; BCR, B-cell receptor; B-NHL, B cell non-Hodgkin lymphoma; CLL, chronic lymphocytic leukemia; CXCR4, CXC chemokine receptor 4 specific for stromal cell-derived factor-1. DLBCL, diffuse large B cell lymphoma; FL, follicular lymphoma; HCL, hairy cell leukemia; MCL, mantle cell lymphoma; MM, multiple myeloma; MZL, marginal zone lymphoma; ROR1, receptor tyrosine kinase-like orphan receptor; SLL, small lymphocytic lymphoma; WM, Waldenström’s macroglobulinemia. Present on precursor B lymphocytes, pre-BCR differs from conventional BCRs in that it possesses a germ line-encoded surrogate light chain which is associated with the signal transduction machinery via heavy chain proteins that have been generated by productive rearrangement of the immunoglobulin HC genes.

## 2. Current Treatments and Clinical Trials of B Cell Malignancies Using Drugs That Target BCL2, BTK, PI3K Proteins, and TAAs (CD19, CD20, CD22, CD38)

**Figure 2:**
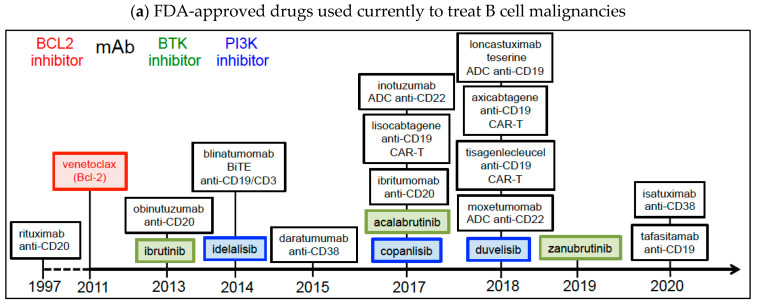

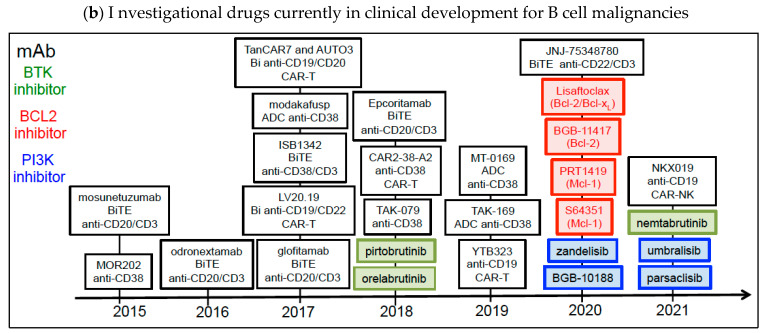
Timeline from 1997 onwards, showing the development of therapeutics for B cell malignancies. (**a**) (1997–2020) Drugs currently approved by the FDA for the treatment of B cell malignancies; (**b**) investigational drugs in clinical development (2013–2021) for B cell malignancies. ADC, antibody–drug conjugate; BCL2, B-cell lymphoma/leukemia 2; BiTE, bispecific T-cell engager, BTK, Bruton tyrosine kinase; CAR-T, chimeric antigen receptor-T cells; PI3K, phosphatidylinositol-3 kinase.

The drugs currently approved by the FDA for the treatment of B cell malignancies [27] still include the first-in-class anti-CD20 agent (rituximab), the first-in-class Bcl-2 inhibitor (venetoclax), the first-in-class BTK inhibitor (ibrutinib), and the first-in-class PI3K inhibitor (idelalisib). Figure 2 charts these key discoveries, which led to the development of more efficacious treatments (Figure 2a) and new indications for clinical evaluation (Figure 2b). These agents include BCL2, BTK, and PI3K inhibitors, together with mAbs and their derivatives (ADC, ARC, bispecific Ab, and CAR-T).

### 2.1. BCL2 Inhibitors

Over the last 20 years, significant efforts have been made to develop the therapeutic potential of agents targeting BCL2 family members [3,30,60]. The broad panel of small molecules developed to mimic the action of anti-apoptotic proteins includes navitoclax/ABT-263 (targeting Bcl-x_L_, Bcl-2, and Bcl-w), obatoclax/GX15-070 (targeting Bcl-2, Bcl-x_L_, Mcl-1, and Bcl-w), and venetoclax/ABT-199 (targeting Bcl-2 only); all have entered clinical trials [3,61,62]. Navitoclax and obatoclax induced severe adverse events, and the trials were stopped [3,61,62]. Venetoclax was approved in 2016 for treatment of previously treated CLL patients with a 17p deletion [3,60]. Resistance to venetoclax appeared related (at least in part) to upregulation of anti-apoptotic members Mcl-1 and Bcl-x_L_, acquired Bcl-2 mutations, and the post-translational phosphorylation of Bcl-2 [61]. Thus, rational combinations of venetoclax with other agents were expected to produce a better clinical response [61]. In 2018, FDA expanded venetoclax’s market authorization so that it could be combined with the anti-CD20 rituximab for the treatment of patients whose CLL had progressed after a single course of treatment, regardless of their 17p deletion status [61]. When combined with rituximab or obinituzumab (also an anti-CD20 agent), venetoclax is now an approved standard of care for treatment- and relapsed CLL disease (Figure 2a and Table 2) [30]. Further, many clinical trials have combined venetoclax with other chemotherapeutic agents in CLL; for recent reviews, see [60,63]. Three ongoing phase II trials are evaluating the venetoclax + ibrutinib combination in treatment-naive CLL patients (NCT02910583) [64] and patients with relapsed/refractory (R/R) CLL/small lymphocytic lymphoma (SLL) (NCT0275689) [65], and the venetoclax + ibrutinib + obinutuzumab combination in treatment-naive CLL patients with a p53 deletion (17p-) and/or mutation (NCT02758665).

Bcl-2 is expressed strongly and constitutively in most B-NHL (Table 1). In this context, venetoclax has also been evaluated in patients with MM, FL, MCL, WM, MZL or even B-ALL or DLBCL (where ≈30% of tumors express high levels of Bcl-2) [3,60]. While venetoclax exhibited marked single-agent activity in MCL and WM, its outcome in treating the other B neoplasms was not as satisfactory [3,60,66]. Many clinical trials have started to evaluate various combination regimens of venetoclax in NHL and B-ALL (reviewed in [3,30,59,60,61,63]). Two phase II/III trials are evaluating the efficacy of venetoclax + ibrutinib in patients with relapsed MCL (NCT03112174) [67] and in WM with a specific MYD88 gene mutation (NCT04273139). In 2018, a phase I clinical trial of venetoclax + lenalidomide + rituximab started in patients with previously untreated MCL (NCT03523975). Unexpectedly, a phase III trial of venetoclax in R/R MM patients receiving bortezomib and dexamethasone as their standard therapy (NCT02755597) was suspended in 2022 [68]. A phase II study (NCT02055820) showed that venetoclax was efficacious and safe when combined with R-CHOP in DLBCL patients expressing high levels of Bcl-2 protein [69]. There are few trials of combinations of venetoclax with other targeted agents in FL, MZL and B-ALL [60]. With regard to B-ALL, the Bcl-2 and Bcl-x_L_ dependence of cell lines and primary cells has prompted clinical trials of BCL2 antagonists as single agents or combination therapies [70,71]. A retrospective study confirmed the safety and efficacy of venetoclax when compared with conventional treatments of ALL in pediatric patients [72]. In a phase I study (NCT03181126), a combination of venetoclax with low-dose navitoclax and chemotherapy was well tolerated in both pediatric and adult patients with R/R B/T-ALL [73]. Two phase I/II trials (NCT03236857, NCT04029688) of venetoclax alone or combined with chemotherapeutics are being initiated for pediatric patients with relapsed B/T-ALL [74,75]. As a whole, these clinical trial data suggest that the addition of venetoclax to conventional chemotherapies improves response rates for NHL and B-ALL.

Treatment with venetoclax can be impeded by frequent gastrointestinal adverse events, neutropenia, and the emergence of specific, resistance-inducing BCL2 mutations [30,60]. Recently, two new Bcl-2 inhibitors were designed and developed: BGB-11417 targets Bcl-2 only, whereas lisaftoclax/APG-2575 targets Bcl-2 and Bcl-x_L_. In phase I trials, both compounds were found to be safe and effective in CLL/SLL and B-NHL (Table 3) [76,77]. These findings warrant further clinical investigation.

The anti-apoptotic protein Mcl-1 is a major factor in resistance to cancer chemotherapies and contributes to the onset of venetoclax resistance in some Mcl-1-overexpressing tumor cells [78]. Since 2012, many Mcl-1 inhibitors have been developed [79,80,81,82,83,84]. AMG176 [81], AMG-397 [82], and AZD5991 [84] were suspended due to the occurrence of serious adverse events in phase I trials for hematological malignancies, while the trial of ABBV-467 in R/R MM was terminated in 2021 for strategic reasons. A phase I study is now evaluating a novel Mcl-1 inhibitor (PRT1419) in patients with R/R MM and NHL (Table 3). Interestingly, the combination of another Mcl-1 inhibitor (S64315, also referred to as MIK665) with azacytidine is starting to be assessed in patients with acute myeloid leukemia (AML) [83]; the clinical value of S64315 in B lymphoid malignancies has yet to be assessed.

**Table 2:** Current treatments of B cell malignancies with FDA-approved drugs targeting Bcl-2, BTK, PI3K and TAAs (CD19/CD20/CD22/CD38), alone or in combination and as first- or second-line therapies.

B Cell Malignancy	Current Treatment Options	References
B-ALL	Conventional chemotherapies * Tyrosine kinase inhibitors imatinib or dasatinib for patients with Philadelphia chromosome-positive **Blinatumomab** **Tisagenlecleucel** **Inotuzumab ozogamycin** (adults)	[85,86,87,88,89]
CLL/SLL	Fludarabine, cyclophosphamide and **rituximab** (FC**R**) Bendamustine and **rituximab** (B**R**) **Rituximab** and chlorambucil (**R**Cb) **Rituximab**, cyclophosphamide, doxorubicin, vincristine, and prednisone (**R**-CHOP) **Obinutuzumab** and chlorambucil **Ibrutinib** or **acalabrutinib**, alone **Idelalisib** alone and with **rituximab** **Venetoclax** + **rituximab** (or **obinituzumab**)	[60,90,91,92,93,94,95,96,97]
HCL	Purine analogs (cladribine, pentostatin) alone and with **rituximab** **Moxetumomab pasudotox**	[98,99]
MM	Conventional chemotherapies ** Daratumumab (or **isatuximab**) + lenalidomide (or pomalidomide) + dexamethasone **Belantamab mafodotin**	[34,49,100,101,102]
FL	B**R** **Rituximab**, cyclophosphamide, vincristine, and prednisone (**R**-CVP) **R**-CHOP **Rituximab** + hyaluronidase human **Rituximab** + lenalidomide **Obinutuzumab** + bendamustine **Idelalisib** or **copanlisib** alone **Ibritumomab** tiuxetan (Yttrium-90) **Axicabtagene ciloleucel**	[103,104,105,106,107]
MCL	**R**-CVP, **R**-CHOP **Rituximab** + lenalidomide **Rituximab** + bortezomib **Ibrutinib** or **acalabrutinib** or **zanubrutinib**, alone **Ibrutinib** + **rituximab**	[53,108,109]
MZL	**R**-CVP, **R**-CHOP, FC**R**, B**R** Fludarabine and **rituximab** (F**R**) **Rituximab** + lenalidomide **Ibrutinib** or **zanubrutinib**	[54,110,111,112]
DLBCL	**R**-CHOP **R**-CHOP + etoposide **Rituximab** **Tafasitamab** + lenalidomide **Loncastuximab teserine** **Tisagenleucel** **Axicabtagene ciloleucel**	[89,113,114,115,116,117]
WM	Alkylating drugs and proteasome inhibitors both in combination with **rituximab** **Ibrutinib** or **acalabrutinib** or **zanubrutinib**, alone and with **rituximab**	[59,118,119,120,121,122]

* Various combinations of vincristine, dexamethasone or prednisone, and an anthracycline drug (doxorubicin or daunorubicin); fludarabine, cytarabine and G-CSF; high-dose cytarabine; cytarabine and mitoxantrone; bortezomib. ** Proteasome inhibitors (bortezomib, carfilzomib, and ixazomib), immunomodulatory drugs (lenalidomide, pomalidomide, and thalidomide), alkylating agents (cyclophosphamide and bendamustine), steroids (prednisone and dexamethasone), a nuclear export inhibitor (selinexor), and bisphosphonates, alone or in various combinations. R-CHOP, rituximab, cyclophosphamide, doxorubicin, vincristine, and prednisone. The FDA-approved drugs listed in Figure 2a are written in bold type.

**Table 3:** Selected active or recruiting clinical trials of novel inhibitors of BCL2/BTK/PI3K proteins or TAAs (CD19/CD20/CD22/CD38), evaluated alone or in combination as first- or second-line therapies for B cell malignancies.

Drug	Class	Disease Setting	Phase	Study	References
BGB-11417	Bcl-2 inhibitor	CLL/SLL, B-NHL	I	NCT04277637 Recruiting, 2020–2023 Alone or combined with zanubrutinib	[76]
Lisaftoclax/ APG-2575	Bcl-2/Bcl-x_L_ inhibitor	CLL/SLL, MZL, MCL CLL/SLL	I Ib/II	NCT03913949 Recruiting, 2019–2023 NCT04494503 Recruiting, 2020–2023 Alone or combined with rituximab or ibrutinib	[77] [123]
PRT1419	Mcl-1 inhibitor	R/R MM R/R B-NHL	I	NCT04543305 Active, 2020–2022	[124]
S64351/ MIK-665	Mcl-1 inhibitor	AML	I/II	NCT04629443 Recruiting, 2020–2024 + azacytidine	
Orelabrutinib /ICP-022	BTK inhibitor	MCL CLL/SLL FL, MZL, MCL, CLL/SLL R/R MZL, R/R WM, DLBCL	I/II I/II II	NCT03494179 NCT03493217 Active, 2018–2022 NCT04014205 Recruiting, 2019- NCT03797456 NCT04440059 NCT04438005 Recruiting, 2020-	[125]
Nemtabrutinib/ MK-1026/ARQ-531	BTK inhibitor	CLL/SLL, MCL, R/R MZL R/R FL, R/R WM	II	NCT04728893 Recruiting, 2021–2027	
Pirtobrutinib/ LOX-305	BK inhibitor	Previously treated CLL/SLL, WM, MCL, MZL Untreated CLL/SLL Previously treated CLL/SLL	I/II III III	NCT03740529 Recruiting, 2018–2023 Combined with venetoclax + rituximab NCT05023980 Recruiting 2021- NCT04965493 Recruiting, 2021- Combined with venetoclax + rituximab	
Parsaclisib/INCB500465/ IBI-376	PI3Kδ inhibitor	CLL, DLBCL	I/II	NCT04809467 Recruiting, 2021–2023 Alone or combined with tafasitamab	
Zandelisib/PWT143/ME-401	PI3Kδ inhibitor	R/R FL, R/R MZL R/R CLL	III II	NCT04745832 Recruiting, 2021–2031 Combined with rituximab NCT05209308 Recruiting, 2022–2026 Combined with rituximab + venetoclax	[126]
BGB-10188	PI3Kδ inhibitor	R/R CLL, FL, MCL, DLBCL, MZL	I/II	NCT04282018 Recruiting, 2020–2025 Alone or combined with zanubrutinib and tiselizumab	
Umbralisib/ TGR-1202	Dual PI3Kδ/CK1ε inhibitor	R/R CLL, R/R MCL R/R CLL, R/R B-NHL	I/Ib I	NCT02268851 Active, 2014–2023 Combined with ibrutinib NCT03283137 Active, 2017–2024 Combined with pembrolizumab	[127]
Mosunetuzumab/ BTC4465A	BiTE anti-CD20/CD3	CLL, B-NHL B-NHL	I/II I/II	NCT02500407 Recruiting, 2015- Alone or combined with atezolizumab (anti-PD-L1) NCT03671018 Recruiting, 2018- Alone or combined with polatuzumab (anti-CD79B-monomethyl auristatin E)	[128]
Odronextamab/ REGN1979	BiTE anti-CD20/CD3	R/R DLBCL, FL, MZL, WM	I/II	NCT02651662 Active, 2016–2026 Alone or combined with cemiplimab (anti-PD-1) NCT03888105 Recruiting, 2019–2028	[129]
Epcoritamab/ GEN301	BiTE duoBody-anti-CD20/CD3	R/R or progressive DLBCL, MCL, FL, MZL, SLL R/R DLBCL R/R CLL	I/II III I/II	NCT03625037 Recruiting, 2018–2024 NCT04628494 Recruiting, 2020–2024 NCT04623541 Recruiting, 2020–2024	[130,131]
Glofitamab (RO7082859)	BiTE anti-CD20/CD3	R/R B-NHL	I/II Ib/II	NCT03075696 Recruiting, 2017–2023 Alone or combined with obinutzumab NCT03533283 Recruiting, 2018–2024 Alone or combined with atezolizumab, obinutuzumab, tocilizumab polatuzumab vedotin	[132]
Tafasitamab/ MOR208/	Anti-CD19	R/R FL, R/R MZL DLBCL	III III	NCT04680052 Recruiting, 2020- Combined with rituximab + lenalidomide compared with rituximab + lenalidomide NCT04824092 Recruiting, 2021–2026 Combined with lenalidomide + R-CHOP	[133]
CAR-20.19-T	Anti-CD19/CD20 CAR-T/CD28/ 4-1BB/CD3ζ	CLL/SLL, R/R B-NHL	I	NCT03019055 Active, 2017–2022	[134]
CD19-CAR-NK using blood cord NKs	Anti-CD19 CAR-NK/OX40/CD3ζ/IL-15	B-ALL CLL/SLL, R/R B-NHL	I/II	NCT03056339 Active, 2017–2022 after treatment with fludarabine/cyclophosphamide lymphodepletion	[135,136]
NKX019 using peripheral blood NKs	Anti-CD19 CAR-NK/OX40/ CD3ζ/IL-15	B-ALL CLL/SLL, R/R B-NHL	I	NCT05020678 Recruiting 2021–2023	[137]
C-CAR066/CBM.20-CAR-T	Anti-CD20 CAR-T	NHLs resistant to rituximab or anti-CD19 CAR-T	I	NCT04036019 Recruiting, 2019–2021 NCT04316624 Recruiting, 2020-	[138]
Inotuzumab ozogamicin	ADC anti-CD22- calicheamicin	B-ALL, B-NHL	III	NCT03959085 Recruiting, 2019–2029 post chemotherapy	
JNJ-75348780	BiTE anti-CD22/CD3	CLL, B-NHL	I	NCT04540796 Recruiting, 2020–2024	
CD22-CAR	Anti-CD22 CAR-T/CD8/4-1BB/CD3ζ	R/R B-ALL, B-NHL	I	NCT02315612 Recruiting, 2014–2040	[139]
LV20.19	Bi anti-CD19/CD22 CAR-T/4-1BB/CD3ζ	CLL/SLL, B-NHL	I	NCT03019055 Active, 2017–2022	[134,140]
AUTO3	Bi anti-CD19 (OX40 costim)/CD22 (4-1BB costim) CAR-T	R/R DLBCL	I/II	NCT03287817 Active 2017-	[141]
CD19-22.BB.z-CAR	Bi anti-CD19/ CD22 CAR-T	R/R B-ALL, DLBCL Pretreated B-ALL	I I	NCT03233854 Recruiting, 2017–2025 Combined with NKTR-255 (IL-15 receptor agonist) NCT03448393 Recruiting, 2018–2040 following cyclophosphamide/fludarabine treatment	[142] [143]
CD20-22 CAR	Bi anti-CD20/CD22 CAR-T	B cell malignancies	I	NCT04283006 Recruiting, 2020–2028	
Felzartamab/ MOR202/TJ202	Anti-CD38	R/R MM	II III	NCT03860038 Active, 2019–2022 Combined with dexamethasone NCT03952091 Active, 2019–2022 Combined with lenalidomide + dexamethasone	
TAK-079	Anti-CD38	R/R MM MM	I/IIa I	NCT03499280 Completed, 2018–2022 Alone or combined with pomalidomide and dexamethasone NCT03984097 Active, 2019–2023 Combined with lenalidomide + dexamethasone	
TAK-573/ modakafusp	ADC anti-CD38 delivering attenuated IFN-α2b	R/R MM	I/II	NCT03215030 Recruiting, 2017- Alone or combined with dexamethasone	[144]
TAK-169/ MT-0169	ADC anti-CD38 delivering Shiga-like toxin	R/R B-NHL	I	NCT04017130 Recruiting, 2019-	[145]
ISB 1342/ GBR 1342	BiTE anti-CD38/CD3	R/R MM	I	NCT03309111 Recruiting 2017–2024	

### 2.2. BTK Inhibitors

Of the many BTK inhibitors developed in the last 10 years, three (ibrutinib/PCI-32765, acalabrutinib/ACP 196, and zanubrutinib/BGB 3111) are now used in the clinic to treat B cell malignancies (Figure 2a and Table 2). Ibrutinib and acalabrutinib are authorized for untreated and R/R CLL/SLL and CLL with 17p deletion [146,147,148,149], ibrutinib and zanubrutinib are authorized for R/R MZL patients who have received at least one line of an anti-CD20-based regimen [149], and ibrutinib, acalabrutinib and zanubrutinib are authorized for WM patients and for MCL patients who have received at least one line of treatment [119,147,148,149,150]. These three agents are orally administered alone or in combination with an anti-CD20 mAb such as rituximab (Table 2). Acalabrutinib and zanubrutinib are better tolerated than ibrutinib, although the first two drugs have not been on the market as long as the latter [149]. All three drugs inhibited BTK irreversibly by forming a covalent bond with cysteine-481 (Cys-481) in the active site [28,151]. The first-generation BTK inhibitor ibrutinib has off-target kinase inhibition activities [28], whereas the second-generation BTK inhibitors acalabrutinib and zanubrutinib are more selective for BTK [28,149].

Several completed phase I trials have demonstrated the safety of ibrutinib and acalabrutinib in R/R MM, either alone or combined with other therapeutics [152]. A phase III trial (NCT01855750) showed a survival benefit of the addition of ibrutinib to R-CHOP chemotherapy in younger patients with DLBCL [153], while a phase II study (NCT01779791) did not find any benefit of ibrutinib in R/R FL [154]. In a phase III trial (NCT03734016), zanubrutinib significantly improved response rates and delayed disease progression in patients with R/R CLL/SLL (relative to ibrutinib) and did so with less toxicity [155]. The efficacy and safety of zanubrutinib are being evaluated in treatment-naive CLL/SLL patients with and without a 17p deletion (NCT03336333). Adding zanubrutinib to obinutuzumab improves responses and progression-free survival in R/R FL (NCT03332017, 2017–2023) [156]. In a phase II study (NCT03145064), zanubrutinib monotherapy led to modest responses in R/R DLBCL patients with both CD79B and MYD88 mutations [157].

The resistance of lymphoid tumors to treatment with first- and second-generation BTK inhibitors and subsequent relapse appear to be in part related to the acquisition of mutations in BTK [149,158]. The most common is the Cys-481 to serine (C481S) mutation, which disrupts inhibitor-BTK binding [149]. To overcome this resistance problem, third-generation noncovalent BTK inhibitors have been developed to inhibit the kinase activities of both BTK and BTK^C481S^ [28,149]. These drugs include vecabrutinib/SNS-062, fenebrutinib/GDC-0853, orelabrutinib/ICP-022, nemtabrutinib/MK1026/ARQ-531, and pirtobrutinib/LOX-305. The completed phase I trials with vecabrutinib in CLL/SLL and B-NHL (NCT03037645) and with februnitib in R/R NHL and CLL (NCT01991184) showed that these drugs were well tolerated but exhibited low levels of antitumor activity; hence, the studies were discontinued [159]. Orelabrutinib, pirtobrutinib, and nemtabrutinib are undergoing clinical development as monotherapies or combination therapies in most B cell malignancies (Table 3).

### 2.3. PI3K Inhibitors

PI3K isoforms (PI3Kα, PI3Kβ, PI3Kδ and PI3Kγ) are commonly found in B cell malignancies and are activated by BCR signaling [160,161]. This is why targeting the PI3K pathway has promise in treating B cell malignancies. At present, two PI3K inhibitors are used in routine clinical practice for the treatment of patients with CLL and FL: idelalisib (an orally administered PI3Kδ inhibitor) and copanlisib (an intravenously administered pan-PI3K inhibitor) [162,163] (Figure 2a and Table 2). As the first-in-class PI3Kδ inhibitor, idelalisib showed efficacy in patients with a 17p deletion and p53 mutation, unmutated IGHV status, and R/R CLL [161]. The drug is currently used in combination with rituximab (Table 2) [164]. Idelalisib and copanlisib have been approved for the treatment of R/R FL in patients having received two or three lines of treatment (Table 2) [107,164,165,166]. A recent, retrospective, Italian multicenter study confirmed the effectiveness of idelalisib in R/R FL patients treated in routine clinical practice [167]. Compared with idelalisib, copanlisib was associated with a higher complete remission rate and a more favorable safety profile [107]. It should be noted that the oral dual PI3Kδ/γ inhibitor duvelisib (initially approved in 2018 for the treatment of patients with R/R FL having received at least lines of systemic treatment [168]) was withdrawn from the market by its developer Secura Bio in December 2021, following the occurrence of fatal and/or serious infections [169].

Despite the recent withdrawal of duvelisib in the indication of FL, several clinical trials in CLL/SLL are still evaluating duvelisib as combination treatments and with different dosing regimens [161,162]. A phase III trial (NCT02004522) of duvelisib vs. ofatumumab (an anti-CD20) in patients with R/R CLL/SLL was terminated in 2021 after the occurrence of serious adverse events, including severe infections and diarrhea/colitis [161]. Clinical evaluations of the efficacy of idelalisib as a monotherapy and of duvelisib combined with anti-CD20 (obinutuzumab) in R/R MM were recently reviewed by Grimont et al. [119]. Idelalisib is associated with significant hepatotoxicity, which may limit the drug’s use in WM [118]. Four phase I/II studies are evaluating copanlisib as part of a combination therapy for CLL, FL, MZL and DLBCL (NCT03884998), DLBCL (NCT03484819), untreated FL (NCT03789240), and DLBCL and R/R grade 3b FL (NCT03789240) (reviewed in [107,164]). In an ongoing phase III trial (NCT02367040), copanlisib was found to be safe when combined with rituximab for the treatment of R/R NHL [170].

Given the potentially limiting toxicities reported for idelalisib and copanlisib [161], further improvements are required to develop new, safer, and more selective PI3K inhibitors. Many PI3K inhibitors have been developed over the last five years, and some of them are being tested in the clinic as monotherapies or combination therapies (reviewed in [12,160,161]). For example, the oral PI3Kδ inhibitor parsaclisib (used alone or in combination with chemotherapy) demonstrated antitumor activity and an acceptable safety profile in R/R B-NHL (the phase II NCT02998476 and phase III NCT02018861 both completed in 2021) [171,172]. Following a business decision in February 2022, Incyte withdrew parsaclisib as a treatment for patients with R/R MCL, MZL and FL [173]. However, a combination of parsaclisib with the anti-CD19 mAb tafasitamab is still being evaluated in patients with R/R-CLL and DLBCL (Table 3). Furthermore, two potent, selective PI3Kδ inhibitors (zandelisib and BGB-10188) are in clinical development for the treatment of patients with R/R CLL and B-NHL. Zandelisib (alone or in combination with rituximab) was found to be well tolerated in all R/R neoplasms CLL/SLL, R/R FL, MZL and DLBCL (NCT02914938) [174]. A multicenter, randomized phase III study is now investigating the safety and efficacy of the zandelisib + rituximab combination vs. standard immunochemotherapy in patients with R/R FL or MZL (Table 3) [126], while a phase II trial has been initiated with a zandelisib + rituximab + venetoclax combination in patients with R/R CLL (Table 3). In a phase I/II study, BGB-10188 is being evaluated as a monotherapy and a combination therapy with zanubrutinib and tislelizumab (an anti-PD1) in patients with CLL and B-NHL (MCL, FL, DLBCL, and MZL) (Table 3). Lastly, umbralisib/TGR1202 inhibits both PI3Kδ and casein kinase 1ε (CK1ε); the latter is involved in the translation of the c-Myc oncogene and the regulation of the Wnt pathway [175]. Umbralisib is associated with less immune-mediated toxicity than the other PI3Kδ inhibitors (because it disables WNT signaling by inhibiting CK1ε) and exerts less detrimental effects on regulatory T cells’ immunosuppressive functions [175]. Umbralisib was found to be efficacious and safe as a monotherapy for patients with R/R CLL and B-NHL [176] and for CLL patients who do not tolerate BTK or PI3Kδ inhibitor therapy [177]. The drug’s favorable toxicity profile was confirmed when it was combined with ibrutinib in patients with R/R CLL and MCL [127] or with ublituximab (an anti-CD20) in patients with CLL/SLL and B-NHL [178]. In January 2022, however, the FDA placed a partial clinical hold on studies of umbralisib and ublituximab [160,161] in an indication of CLL or B-NHL [179]. Studies of a combination of umbralisib with ibrutinib or pembrolizumab (an anti-PD1) in patients with R/R CLL and R/R B-NHL are ongoing (Table 3).

### 2.4. mAbs and CAR-T Cells

Antibody-based cancer treatments have developed quickly over the past 25 years. Following on from the first generation of therapeutic murine mAbs (-momabs), a variety of second-generation mAbs were engineered: chimeric mAbs (-ximabs), humanized mAbs (-zumabs), and human mAbs (-umabs) [180]. Chimeric mAbs possess murine variable regions, whereas the rest of the Ab is of human origin. Humanized mAbs retain murine hypervariable segments, whereas fully human mAbs have a native Fc region [180]. Third-generation mAbs have an engineered Fc region, which is designed to improve therapeutic performance by adapting their effector functions [180]. The main effector pathways commonly employed by mAbs are complement-dependent cytotoxicity (CDC), Ab-dependent cellular cytotoxicity (ADCC), antibody-dependent cellular phagocytosis (ADCP), and programmed cell death (PCD) [181,182].

To enhance their therapeutic value, mAbs have also been conjugated with toxins (giving ADCs), radioisotopes (giving ARCs), immunomodulatory cytokines, and costimulatory molecules (giving CAR-T cells) [180,182,183,184,185]. An ADC combines selectivity and cytotoxic potency and so can target a given TAA with high specificity [183,186]. After binding to a TAA, ADCs enter the cell via receptor-mediated endocytosis and release the lethal drug. ADCs can also activate ADCC and ADCP signaling by immune effector cells [186]. Multivalent Abs simultaneously target a TAA and an activating receptor on the effector cell (typically a T cell) [187]. Most engineered, multispecific Abs lack an Fc region and therefore do not mediate CDC or ADCC [184]. Bispecific T-cell engagers (BiTEs) are bispecific, single-chain Abs that specifically recognize the T cell antigen CD3 and a TAA, which also stimulate cytotoxic T cells for targeted tumor cell lysis [184,185]. CAR-T therapy is based on antibody recognition of TAAs [188,189]. CAR-T cells are cytotoxic T cells that have been modified genetically to express an artificial chimeric antigen receptor (CAR). The CAR comprises an extracellular target-binding domain (composed of a single-chain variable fragment (scFv) from antibody light- and heavy-chain variable regions and which bind to the TAA), a spacer/hinge region, transmembrane domains, and intracellular domains (comprising two or more costimulatory domains (CD28 and/or 4-1BB/CD137/TNF-RSF9) and a T cell signaling domain (with CD3ξ) [14,190,191]. The first generation of CAR constructs included CD3ξ; the second generation included CD28 and CD3ξ, or 4-1BB and CD3ξ; the third generation included CD28, 4-1BB, and CD3ξ; the fourth generation included a cytokine transgene initiated through NFAT signaling, and the fifth generation included IL-2Rβ (to initiate JAK/STAT signaling) [14,190,191]. Following expansion in the laboratory and then infusion into the patient, CAR-T cells bind to their cognate targets on tumor cells through their extracellular domain and initiate signal transduction that triggers the effector functions of host cytotoxic T cells, which then kill the tumor cells [188,189].

#### 2.4.1. CD20 mAbs and CD20-Targeting CAR-T

Within the last 20 years, a variety of strategies have been used to develop a large number of anti-CD20 mAbs. The FDA’s approval of rituximab in 1997 marked a breakthrough in the treatment of B cell malignancies (Figure 2a). Rituximab is still part of the standard of care for most B cell tumors (Table 2). Rituximab engages the main effector pathways employed by mAbs (i.e., CDC, ADCC, and ADCP) and even directly activates PCD [182,192]. Many phase I/II/III trials have evaluated the combination of other novel drugs (including ibrutinib, idelalisib, acalabrutinib, venetoclax, ipilimumab (an anti-CTLA4), chromatin modulators, and other drugs) with rituximab in B cell malignancies [15,193]. Based on the efficacy, relative safety and ubiquitous use of rituximab in B cell malignancies, attempts have been made to develop more efficacious and/or less toxic CD20 mAbs [15,184,185,194,195]. Indeed, the CD20 mAbs obinutuzumab and ibritumomab tiuxetan (radiolabeled with ^90^Yttrium) are now used to treat CLL and FL (Figure 2a and Table 2), and four BiTE anti-CD20/CD3 mAbs (mosunetuzumab, odronextamab, glofitamab and epcoritamab) are in clinical development (Figure 2b and Table 3) [128,130,131].

Obinutuzumab’s mechanisms of action are similar to those of rituximab [182,195]. ^90^Y-ibritumomab binds to the CD20 antigen on tumor B cells, and the long range of β particles from the ^90^Y means that neighboring tumor cells can be killed without direct antibody binding [195]. Compared with rituximab in the induction therapy of B-NHL, obinutuzumab was associated with significantly greater progression-free survival but a greater incidence of adverse events, and ^90^Y-ibritumomab was associated with a greater overall response rate [33].

Mosunetuzumab, odronextamab and glofitamab monotherapies have already show manageable safety profiles and given lasting responses in heavily pretreated patients with R/R B-NHL (Table 3) [128,132,196]. Compared with intravenously infused mosunetuzumab, odronextamab or glofitamab, the subcutaneously administered epcoritamab is usefully associated with (i) lower peak cytokine levels, (ii) a lower treatment burden for patients, and (iii) less resource use at the treatment facility [130,131]. Mosunetuzumab is currently in phase I/II trials, where it is combined with mAbs targeting the programmed cell death protein 1 (PD-1) immune checkpoint, the latter’s ligand (programmed death-ligand 1, PD-L1), or CD79B (an antigen associated with the BCR; see Figure 1b) (Table 3). Other novel BiTE anti-CD20/CD3 mAbs are being evaluated in phase I trials [184].

Several clinical studies have focused on the potential of autologous, first-generation anti-CD20 CAR-T cell therapies in patients with B cell malignancies (reviewed in [138,197,198]). Two phase I clinical trials are underway to evaluate the efficacy of C-CAR066 (a novel, second-generation, anti-CD20 CAR-T cell therapy) in patients with R/R NHL (including cases of DLBCL) after the failure of rituximab or CD19 CAR-T therapy (Table 3) [138]. All the enrolled B-NHL patients who were previously R/R to rituximab achieved various clinical responses, and the level of toxicity was tolerable [138].

#### 2.4.2. CD19 mAbs and CD19-Targeting CAR-T

Many models based on CD19 mAbs have been built and evaluated in the treatment of B cell malignancies [89,199,200]. Several strategies have been implemented, and four types of CD19 mAbs are currently used to treat B-ALL, DLBCL and FL (Table 2): blinatumomab (a BiTE), tafasitamab (an Fc–engineered and Fab affinity-matured Ab), loncastuximab tesirine (ADC), and CAR-T therapies (lisocabtagene maraleucel, axicabtagene ciloleucel and tisagenlecleucel) (Table 2) [89,201,202,203,204,205,206].

By binding both CD19 on B cells and CD3 on T cells, blinatumomab triggers ADC-dependent cytotoxicity [89]. As the first FDA-approved BiTE for the treatment of B-ALL, blinatumomab (developed by Amgen) induces remissions in cases of R/R B-ALL that had failed to respond to other therapies (Table 2) [201,207]. However, blinatumomab has a short half-life and must be continuously infused intravenously [201]. Of the many other CD19 mAbs developed [199], tafasitamab (MOR208) is combined with lenalidomide in the treatment of R/R DLBCL (Table 2) [115,116]. Tafasitamab mediates both tumor B cell apoptosis, ADCC and CDC [208,209]. The main adverse event associated with tafasitamab is bone marrow suppression, with anemia, neutropenia, and thrombocytopenia [115]. At present, two phase III clinical trials are recruiting patients; a combination of tafasitamab and first line-chemotherapeutic agents will be used to treat DLBCL (NCT04824092) [14,89,133] or R/R FL and R/R MZL (NCT04680052) [14,89,133]. Loncastuximab tesirine (ADCT-402) is a humanized CD19 mAb linked to teserine (SG32499); lysosomal degradation delivers a pyrrobenzodiazepine dimer that crosslinks the tumor cell’s DNA [117,202]. Based on the phase II LOTIS-2 trial, loncastuximab tesirine was recently granted fast-track approval for the therapy of adult patients with R/R DLBCL and who have already received two or more lines of treatment (Table 2) [210]. An ongoing phase I clinical trial (NCT03684694) is investigating a combination of loncastuximab and ibrutinib in the treatment of patients with DLBCL and MCL.

The three CD19-CAR-T cell therapies on the market (tisagenlecleucel, axicabtagene ciloleucel, and lisocabtagene maraleucel) use autologous T cells [89,203,205,211]. CAR-T engagement of CD19^+^ tumor cells results in T-cell activation and proliferation, the secretion of inflammatory cytokines and chemokines, and thus tumor cell lysis [212]. Tisagenlecleucel is composed of an anti-CD19 scFv, a CD8-α hinge region, 4-1BB, and CD3ζ [213]. Axicabtagene ciloleucel contains an anti-CD19 scFv, an IgG4 hinge region, CD28, and CD3ζ [212]. Lisocabtagene maraleucel contains an anti-CD19 scFv, an IgG4 hinge region, and CD28, 4-1BB and CD3ζ domains [214]. Tisagenlecleucel has been approved for the treatment of children and young adults with R/R B-ALL (Table 2) [88,213,215,216,217]. In recent phase II/III studies of pediatric/young adult patients with Down-syndrome-associated ALL (NCT02435849, NCT02228096, and NCT03123939), tisagenlecleucel treatment was associated with high remission rates, manageable side-effects, and promising long-term outcomes [216]. From the third line of treatment onwards, both tisagenlecleucel and axicabtagene ciloleucel are currently recommended for patients with DLBCL who have failed autologous transplantation or have relapsed after two previous lines of treatment (Table 2) [32,212,215]. A recent retrospective evaluation of the safety and efficacy of axicabtagene ciloleucel and tisagenlecleucel in DLBCL outside the setting of a clinical trial found that although the two cohorts did not differ significantly with regard to any of the baseline characteristics, cytokine release syndrome and neurologic events were more frequent in the axicabtagene group [218]. An ongoing phase II trial is looking at tisagenleucel CAR-T cell therapy for FL (NCT04094311, 2019–2023). At present, there are three active phase II clinical trials evaluating axicabtagene alone in FL (NCT03105336) [219] and in combination with first line-chemotherapeutic agents (cyclophosphamide/fludarabine) in DLBCL (NCT03761056, NCT03391466) [220]. In 2022, lisocabtagene was approved as a second-line treatment of patients with R/R DLBCL and FL and who were not eligible (due to comorbidities or age) for hematopoietic stem cell transplantation (Table 2) [214]. Two phase I/II studies will evaluate the efficacy and safety of lisocabtagene in combination with other chemotherapeutic agents in R/R CLL/SLL (NCT03331198, with ibrutinib or venetoclax) [221], and high-risk DLBCL and FL (NCT03310619: lisocabtagene alone or combined with ibrutinib or durvalumab). Relative to standard care, the lisocabtagene + ibrutinib combination yielded significantly higher event-free survival and response rates in patients with R/R CLL/SLL [221]. Thus, CD19-CAR-T therapy appears to have promising outcomes and a tolerable safety profile in R/R B cell malignancies [222,223]. Further modifications of the CAR’s structure (resulting in second/third-generation anti-CD19/CAR-T agents) and the therapeutic strategy are being tested as treatments for B cell malignancies (reviewed in [31,223,224]).

Given that the emergence of CD19^-^ tumor cells substantially increases the risk of relapse [32], another approach seeks to optimize single-chain, bispecific CARs (biCARs) that trigger robust cytotoxicity against tumor cells expressing either CD19 or CD20 [134,225,226]. Dual targeting may improve response rates and sustained responses while limiting antigen escape [134,225,226]. In the first-in-human biCAR phase I/IIa trial (NCT03097770, completed in 2020), a bispecific anti-CD19/CD20 tandem receptor (tanCAR7 with 4-1BB/CD3ζ) elicited a potent, lasting antitumor response in the absence of grade 3 or higher cell-related encephalopathy syndrome in patients with R/R NHL [226]. Similarly, another anti-CD19/anti-CD20 CAR-T (CAR-20.19-T) showed low toxicity and high efficacy as single agent in a phase I study with R/R B-NHL (Table 3) [134].

CAR-engineered natural killer (CAR-NK) cells constitute another CAR-based approach to cancer therapy [227,228]. The first anti-CD19 CAR-NK cell construct was composed of an anti-CD19 scFv, CD28, a costimulatory OX40 domain, CD3ζ, and a membrane-bound form of IL-15 [135]. The addition of IL-15 enhanced the proliferation, persistence and activity of NK cells in preclinical models [135]. In a phase I/II trial, HLA-mismatched anti-CD19 CAR-NK cells derived from cord blood were administered to patients with R/R B-NHL, CLL/SLL and B-ALL after standard fludarabine/cyclophosphamide lymphodepletion (Table 3) [135,136]. The majority of the patients responded to the CAR-NK treatment and did not develop serious adverse events [135,136]. More recently, a similar anti CD19-NK cell therapy (NKX019, using HLA-mismatched anti-CD19 CAR-NK cells obtained from peripheral blood) entered a phase I study as a monotherapy for patients with R/R B-NHL, CLL and B-ALL (Table 3) [137]. NKX019 treatment has already shown long-term NK cell persistence and potent antitumor activity [137]. Additional clinical trials for CAR-NK cells for the treatment of B cell malignancies are being set up [14,228].

#### 2.4.3. CD22 mAbs and CD22-Targeting CAR-T

CD22-targeting mAbs have emerged as promising treatment options with proven therapeutic value [16,21,98]. Two ADC-triggering anti-CD22 mAbs (inotuzumab ozogamicin (CMC544) and moxetumomab pasudotox) have been approved for the treatment of adult patients with B-ALL and R/R HCL, respectively (Table 2) [99,229,230,231,232]. Inotuzumab ozogamicin combines an anti-CD22 mAb with the DNA-binding drug calicheamicin, which leads to DNA breakage and apoptosis [16,21]. Moxetumomab pasudotox is an anti-CD22 scFv conjugated to a truncated 38 kDa fragment of *Pseudomonas* exotoxin A (PE38) [233]. In a phase I/II study (NCT01371630, 2011–2025), a combination of inotuzumab ozogamicin with mini-hyperfractionated cyclophosphamide, vincristine, dexamethasone and (in some cases) blinatumomab was found to be highly effective in patients with B-ALL after their first relapse [234]. A phase II study (ITCC-059; EUDRACT 2016-000227-71; NTR5736) provided further evidence of inotuzumab’s activity in pediatric patients with R/R B-ALL [235]; the study is ongoing and is testing a combination of inotuzumab with chemotherapy in pediatric patients with R/R ALL. In an ongoing phase III randomized trial, inotuzumab ozogamicin is being tested as a first-line treatment with high-risk B-ALL and B-lymphoblastic lymphoma (Table 3). However, phase I/II studies of inotuzumab ozogamicin alone or in combination with rituximab for the treatment of B-NHL did not give promising results [236,237,238]. Based on a phase I study of patients with R/R ALL, moxetumomab pasudotox’s clinical activity appears to be moderate [231,239]. Only one phase I study with a BiTE construct targeting CD22 and CD3 (JNJ-75348780) is currently assessing patients with R/R NHL and R/R CLL (Table 3).

Over the last 10 years, many CD22-targeting CARs have been developed and tested in phase I/II clinical trials [16,240]. Two anti-CD22 CARs containing anti-CD22 scFv, IgG4 (or CD8), 4-1BB and CD3ζ domains gave high response rates in patients with B-ALL who had previously failed chemotherapy and/or CD19-targeting CAR-T treatment (Table 3) [139]. Moreover, many bispecific anti-CD19/CD22 CAR-T cells have been developed to prevent relapse by minimizing antigen escape and have been assessed in phase I studies for the treatment of B-cell malignancies [16]. The first anti-CD19/CD22 CAR-T cell construct (4-1BB-CD3ζ, LV20.19 CAR-T) contained a CD19-binding domain derived from a clinically active CD19-CAR, together with a CD22 binding domain [241]. In a phase I trial, LV20.19 showed low toxicity and high efficacy in R/R NHL and CLL (Table 3) [134]. On the same lines, the combination of another dual CD19/22-targeting CAR-T cell therapy (AUTO 3) with pembrolizumab led to complete remission in patients with R/R DLBCL (Table 3) [141]. A new anti-CD19/CD22-CAR-T construct (CD19-22.BB.z, alone or combined with an IL-15 receptor agonist) is being assessed in patients with R/R DLBCL or B-ALL in a phase I trial (Table 3) [142]. According to the initial results, almost all the patients with ALL achieved complete remission [142]. The results of a phase I trial recently confirmed the safety and efficacy of CD19.22.BB.z in heavily pretreated children and young adults with B-ALL (Table 3) [242]. On the basis of these preliminary data, it appears that anti-CD19/CD22 CAR-T cells may improve clinical responses by mitigating target antigen downregulation as a relapse mechanism. Another novel, bispecific anti-CD20/CD22 CAR-T is now being tested for safety in B-cell malignancies (Table 3). A phase I study of monotherapy with anti-CD22-NK cells is planned in patients with R/R NHL and who previously received anti-CD19 treatment (NCT03692767: not yet recruiting).

#### 2.4.4. CD38 mAbs and CD38-Targeting CAR-T

Although the development of therapeutic anti-CD38 mAbs started in the 1990s and has progressed slowly since then, two such mAbs (daratumumab and isatuximab) are now used as combination chemotherapies for patients with R/R MM (Figure 2a and Table 2) [243,244,245,246]. In MM, the two Abs induce ADCC, CDC and ADCP as part of their antitumor mechanism of action [247,248]. Furthermore, isatuximab directly induces both lysosome-dependent death and caspase 3/7-dependent apoptosis of MM cells–even those bearing a p53 mutation [249]. Unlike daratumumab, isatuximab binds to a CD38 epitope that encompasses the CD38 ectoenzyme’s catalytic site, but it is not known whether isatuximab exerts its therapeutic effect by inhibiting CD38’s NADase activity [17]. Usefully, daratumumab is available in formulations for intravenous administration and subcutaneous administration [34,195]. One advantage of isatuximab over daratumumab is the need for less frequent dosing [34,195]. The most common adverse reactions to these two anti-CD38 Abs are neutropenia, pneumonia, upper respiratory tract infection, and diarrhea [34,195].

Following the observation of daratumumab’s and isotuximab’s efficacy in R/R MM, several clinical trials have evaluated the addition of one mAb or the other to standard-of-care regimens in newly diagnosed MM patients [250,251,252]. Based on these studies, the combination of daratumumab with bortezomib/thalidomide/dexamethasone (VRd) was recently approved by the FDA as treatment for these transplant-eligible patients [253]. Since then, two phase III trials (NCT03710603, NCT03652064) have compared the daratumumab-VRd combination with VRd in non-transplant-eligible patients and as an induction treatment before autologous stem cell transplantation in transplant-eligible patients. Other phase III trials are assessing the benefit of combining isatuximab with (i) lenalidomide + bortezomib + dexamethasone or lenalidomide + carfilzomib induction treatment in patients with newly diagnosed MM (NCT03617731, NCT03319667, and NCT04483739), (ii) carfilzomib in patients with R/R MM (NCT03275285, 2017–2023), and (iii) lenalidomide in patients with high-risk smoldering MM (NCT04270409).

Data on the efficacy of daratumumab and isatuximab in other B cell malignancies are scarce. Daratumumab has been tested in phase I trials on patients with CLL and other B-NHL. In CLL, the prognostic value of CD38 expression in patients is well proven (Table 1), and preclinical studies (ex vivo experiments and CLL mouse models) have demonstrated the antitumor efficacy of daratumumab monotherapy or combination therapy [254,255]. The combination of daratumumab with ibrutinib in CLL patients with a poor prognosis (e.g., 17p deletion and/or p53 mutation) is now being studied (NCT03447808 and NCT03734198). The putative efficacy of daratumumab in B-ALL and other types of NHL has not yet been tested. A phase I/II study of the safety and tolerability of a combination of isatuximab with the anti-PD1 cemiplimab in patients with DLBCL started in 2018 (NCT03769181). The fact that CD38 is strongly expressed by some tumor cell subsets in pediatric patients with B-ALL [256] makes this antigen a potential target for the treatment of ALL in children. Isatuximab has significant antitumor activity in vitro and in ALL xenograft models, with robust ADCC- and ADCP-mediated effects [256]. A now-recruiting phase II multicenter study is evaluating the antitumor activity, safety, and pharmacokinetics of isatuximab combined with standard salvage chemotherapies in children after one or two relapses of B-ALL (NCT03860844) [257].

Along with daratumumab and isatuximab, several novel CD38-targeting strategies are being developed (mostly for MM); these include the use of naked anti-CD38 mAbs, anti-CD38 ADCs, anti-CD38/CD3 BiTEs, and CD38-targeting CAR-T cells [195,247,251,258]. Felzartamab (MOR202; TJ202) and TAK-079 are both fully human IgG1 mAbs [251,259]. Although felzartamab showed promising efficacy in MM (both alone and combined with immunomodulators; NCT01421186) [260], the drug’s sponsor (MorphoSys AG) unexpectedly stopped this development in 2020 [259]. However, two ongoing trials are evaluating the effect of a combination of felzartamab and conventional therapies in patients with R/R MM (Table 3). The results of a multicenter phase I study showed that monotherapy with subcutaneously administered TAK-079 is safe and generally well tolerated in patients with R/R MM (Table 3) [261]. Two other clinical trials are evaluating a combination of TAK-079 with polalidomide or lenalidomide + dexamethasone in R/R MM and newly diagnosed MM (Table 3).

Two ADC-triggering anti-CD38 drugs (TAK-573 and TAK-169) have recently entered clinical development in the treatment of R/R NHL (Table 3) [144,145]. TAK-573 (modakafsup) is a CD38 mAb fused to attenuated interferon (IFN)-α; it demonstrates potent anti-MM activity in patients with R/R MM [144]. TAK-169 is an engineered Ab with a deimmunized form of the ribosome-inactivating Shiga-like toxin A subunit genetically fused to a CD38-binding Ab fragment [145]. TAK-169 is internalized by CD38^+^ MM cells, inactivates ribosomes, abrogates protein synthesis and thus kills cells directly [145]. The full results of these clinical trials are eagerly awaited.

Among the many humanized anti-CD38/CD3 BiTEs having been through preclinical development, AMG-424 and ISB 1342 (GBR 1342) have shown strong antitumor effects in MM models [262,263]. A phase I study (NCT03445663) of AMG424 in R/R MM began in 2018 but was terminated by the sponsor for business reasons in 2020. Since ISB 1342 contains a full Fc domain with a reduced effector function, its mechanism of tumor cell killing critically relies on the engagement and activation of T lymphocytes [263]. ISB 1342 is currently in phase I trial for the treatment of patients with MM who have relapsed or who do not respond to standard therapies (e.g., proteasome inhibitors, immunomodulators, and daratumumab) (Table 3).

As is the case for CD19, CD20, and CD22, CD38 is an attractive target for CAR-T therapy. However, given the widespread expression of CD38 (e.g., in plasma cells, precursor B cells, T cells, NK cells, myeloid precursor cells, and various organs), anti-CD38 CAR-T cell constructs can induce various syndromic adverse events [251]. For instance, the administration of anti-CD38 CAR-T cells to a patient with relapsed B-ALL (after the failure of bispecific anti-CD19/CD22 CAR-T cell treatment) was associated with target-mediated toxicity and severe cytokine release syndrome [264]. Some novel anti-CD38 CAR-T cells have been developed with a modified light chain and a high-affinity scFv [251]. A phase I study has evaluated the safety and efficacy of a novel CD38 A2 CAR-T cells construct in patients with R/R MM (NCT03464916, completed in 2022) but it is not known whether this construct has been efficacious in phase II/III trials. The strategies assessed in MM could also be investigated in the treatment of other CD38^+^ lymphoid tumors, including poor-prognosis CLL/SLL, HCL, and other B-NHL. Lastly, the promising preclinical efficacy seen with CD38-targeting NK cells suggests that clinical development is warranted [265,266].

## 3. Clinical Trials of Novel Anti-TAA Inhibitors in the Treatment of B Cell Malignancies

### 3.1. B Cell Maturation Antigen (BCMA) and B-Cell Activation Factor Receptor (BAFF-R)

BCMA (also known as tumor necrosis factor receptor superfamily member 17/TNFRS17) is a transmembrane protein that contains cysteine-rich extracellular domains and lacks a signal peptide [267]. Along with the related TNFR superfamily member BAFF-R, BCMA critically regulates B cell proliferation, survival and differentiation into plasma cells [102,267,268]. BCMA is expressed at significantly higher levels by MM cells (Table 1) than by normal tissues (except for normal plasma cells) [269]. Thus, BCMA has become an important therapeutic target in MM, with four treatment modalities in development: ADCs, BITEs, CAR-T therapies and CAR-NK therapies [102,184,228,269,270,271]. In 2021, the ADC mAb belantamab mafodotin (GSK2857916) was the first anti-BCMA therapy to obtain approval in an indication of R/R MM (Table 2) [272]. Belantamab mafodotin is a humanized anti-BCMA IgG1 mAb linked to the antimitotic drug monomethyl auristatin F [267]. Its efficacy is being evaluated in three clinical trials (Table 4) [273]. MEDI2228 is another ADC, in which an anti-BCMA mAb is covalently linked to the pyrrolobenzodiazepine dimer tesirine. This toxin induces DNA crosslinking and the DNA damage response [274]. The results of a recent phase I study (NCT03489525) [275] demonstrated that MEDI2228 had much the same level of efficacy as belantamab mafodotin [276]. The ADC AMG224 is a BCMA-targeting IgG1 mAb coupled to the tubulin inhibitor mertansine. The results of the first phase I dose-expansion and -escalation study in R/R MM (NCT02561962, 2015–2023) evidenced significant thrombocytopenia [277]. Two BiTE anti-BCMA/CD3 agents (teclistamab and elranatamab) are currently being evaluated in as monotherapies and in combination with other treatments (Table 4) [278]. The initial results for the anti-BCMA CAR-T therapy idecabtagene vicleucel showed clinical activity and the expected adverse effects in patients with R/R (Table 4) [279]. Accordingly, the anti-BCMA CAR-T therapy ciltacabtagene autoleucel was associated with significantly better outcomes than conventional therapies in patients with R/R MM (NCT033548207) [280]. In an early phase I study, the first dual-target anti-BCMA/CD38 CAR-T (BM38) gave a high response rate, a low recurrence rate, and a manageable cytokine release syndrome in patients with R/R MM (Table 4) [281]. A now-recruiting phase I/II study is set to evaluate the safety and antitumor efficacy of BCMA-CAR-NK92 cells in patients with R/R MM (Table 4). Three phase I trials are respectively evaluating adverse events associated with the anti-BAFF-R mAb lanalumab in patients with CLL, and another trial is evaluating anti-BAFF-R-CAR-T cells in B-ALL and R/R MCL (Table 4).

### 3.2. Receptor Tyrosine Kinase-like Orphan Receptor (ROR1)

Aberrant expression of ROR1 is seen in CLL/SLL, MCL, and MM [47]. In CLL, ROR1 expression is associated with relatively short treatment-free and overall survival times [297,298]. As a receptor for Wnt5a [298], ROR1 can stimulate ROR1-dependent CLL cell activation of Rho-GTPases [299]. High expression levels of ROR1 and strong ROR1 signaling are associated with venetoclax resistance in CLL [300]. A phase I/II study in newly treated or R/R CLL patients showed that the mAb anti-ROR1 cirmtuzumab (recently renamed as zilovertamab) was safe and efficacious in inhibiting tumor cell ROR1 signaling in the context of R/R disease (NCT02860676, NCT02222688) [301]. Since then, several clinical trials of a combination of zilovertamab with venetoclax or ibrutunib have been initiated in patients with CLL/SLL or MCL (Table 4).

### 3.3. Program Cell Death 1 (PD1) and PD-Ligand 1 (PD-L1)

The immunosuppressive costimulatory signal receptor PD-1 is expressed on activated T and B cells [302,303]. PD-L1 is strongly expressed by various solid tumors, B tumor cells (including DLBCL and MM cells), and BM stromal cells [303,304]. As the tumor develops, both PD-1 and PD-L1 cause tumor immune escape–mainly by inhibiting the activity of tumor-infiltrating lymphocytes [303,304]. Several mAbs against PD1 (nivolumab, pembrolizumab, and cemiplimab) or PD-L1 (e.g., atezolizumab and durvalumab) have been approved for the treatment of various solid tumors [305,306]. Anti-PD-1 mAbs promote tumor cell apoptosis by binding to T-cell PD-1 receptors and disrupting the interaction with PD-L1 molecules on tumor cells [302]. However, no significant objective responses were observed in two phase I/Ib trials of nivolumab as a monotherapy or a combination therapy in patients with R/R MM [307,308]. Pembrolizumab exhibits selective efficacy in patients with B-NHL and CLL [283], and is currently being tested in combination with ibrutinib or fludarabine (Table 4). In a phase I/II study, a pembrolizumab + blinatumomab combination had a good safety profile (manageable adverse events) in R/R B-ALL (Table 4) [284]. However, a phase III study of a combination of pembrolizumab with pomalidomide and dexamethasone in R/R MM (NCT02576977) found a unfavorable risk-benefit ratio and was terminated [170,309]. Three clinical studies are evaluating the safety and tolerability of cemiplimab in combination with odronextamab (an anti-CD20/CD3 BiTE) or with isatuximab in patients with B-NHL (including R/R MM and DLBCL) (Table 4) [285]. A phase I study of atezolizumab alone or combined with axicabtagene ciloleucel showed a manageable safety profile in patients with R/R DLBCL and thus supported a move into phase II (Table 4) [286]. Durvalumab as a monotherapy or a combination therapy with tremelimumab (an anti-CTLA-4 mAb) or danvatirsen (AZD9150, an antisense oligonucleotide that inhibits STAT3) was well tolerated but showed limited efficacy in R/R DLBCL or FL (NCT02733042) [310,311]. The combination of durvalumab with JCAR014 (CD19/4-1BB CAR-T cells) for the treatment of patients with aggressive B-cell NHL appeared safe; although complete responses were observed in patients at initial restaging after JCAR014 infusion, and in patients continuing durvalumab therapy after initially failing to achieve complete remission (NCT02706405), the study was terminated in 2021 due to slow accrual [312].

### 3.4. C-X-C Chemokine Receptor Type 4 (CXCR4)

CXCR4 is the natural chemokine receptor specific for CXCL12, also termed stromal cell-derived factor-1 (SDF-1), which is released by the stromal cells residing in the thymus and in the bone marrow [313]. The overexpression of CXCR4 in solid and hematological tumors is related to disease progression [41]. In hematological malignancies, high expression of CXCR4 is observed in B cell malignancies (Table 1) [313]. Highly recurrent somatic mutations in *CXCR4* gene are found in up to 40% of patients with WM [313]. Alterations in *CXCR4* are associated with activation of PI3K and JAK/STAT signaling processes, which promote drug resistance by impacting BTK-inhibitor response [313]. Thus, WM patients with mutated *CXCR4* are less likely to respond to ibrutinib or ibrutinib/rituximab combination therapy than WM patients with *CXCR4* wild-type disease [287]. Ulocuplumab (BMS-936564) is a fully human IgG4 anti-CXCR4 mAb [314] that exhibits antitumor activity in a WM xenograft model [315]. A phase Ib/II trial showed that ulocuplumab is safe and leads to a high response rate in combination with lenalidomide and dexamethasone in patients with R/R MM (NCT01359657) [316]. An ongoing phase I/II clinical trial evaluating the combination of ibrutinib and ulocuplumab, in patients with WM, already shows that ulocuplumab dose-escalation did not impact adverse events (Table 4) [287]. Interestingly, an ongoing multicenter phase I trial is assessing the safety and tolerability of mavorixafor (AMD070, an orally bioavailable CXCR4 allosteric antagonist) in combination with ibrutinib in WM patients with *CXCR4* mutation (Table 4).

### 3.5. CS1/Signaling Lymphocyte Activation Molecule Family 7 (SLAMF7)

CS1/SLAMF7 is overexpressed in MM cells, NK cells, plasma cells, and (to a lesser extent) some subsets of B and T cells [317]. Elotuzumab is a humanized IgG1 Ab that directly activates NK cells through the CS1 pathway and Fc receptors, thus facilitating NK-mediated ADCC [317]. In 2015, the US FDA approved elotuzumab in combination with lenalidomide and dexamethasone for the treatment of patients with MM who received one to three prior therapies, with the following warnings and precautions: infusion reactions, infections, second primary malignancies, hepatotoxicity, and interference with the determination of complete response [318]. Two phase I clinical studies are evaluating two different CS1-CAR-T constructs in patients with R/R MM (Table 4).

### 3.6. Other TAAS

#### 3.6.1. CD13

The cell surface protease aminopeptidase N (APN/CD13) is overexpressed in various tumors including hematological diseases [319,320]. Based on its ability to bind to the Asn-Gly-Arg (NGR) motif, CD13 has proven to be a key for targeted delivery of NGR-bound chemotherapeutic drugs to both CD13^+^ tumor cells and CD13^+^ tumor-associated endothelium [320]. Among drugs coupled to the NGR motif, is the anti-angiogenic truncated tissue factor (tTF) that targets CD13^+^ tumor-associated vasculature [320]. A recent phase I trial with NGR-tTF was initiated for patients with CD13^+^ R/R MM (NCT02902237, completed in 2020) indicating that NGR-tTF is safely applicable with this regimen; imaging showed selective reduction of tumor blood flow and intratumoral hemorrhage and necrosis [321]. This innovative approach deserves to be pursued. A second therapeutic strategy exploited the enzymatic activity of CD13 for the activation of the alkylating prodrug melflufen (J1; dipeptide consisting of melphalan and *p*-fluoro-L-phenylalanine) into an active cytotoxic drug, melphalan [322]. In phase I/II studies, melflufen plus dexamethasone demonstrated encouraging clinical activity and a manageable safety profile in heavily pretreated patients with R/R MM (NCT02963493, completed in 2021) [323]. A randomized phase III study is evaluating the efficacy and safety of melflufen/dexamethasone vs. pomalidomide/dexamethasone (Table 4) [288]; melflufen plus dexamethasone shows clinically meaningful efficacy and a manageable safety profile in patients with heavily pretreated R/R MM, including those with triple-class-refractory and extramedullary disease (Table 4) [288].

#### 3.6.2. CD16

NK cells express a CD16 (FcγRIII) V158 variant, which can exert ADCC by binding to the Fc fragment of Ig present on tumor cells [223]. Based on it, one approach was the engineering of T cells expressing CD16 CARs so that they are capable of mediating ADCC [324]. The constructs included the high-affinity CD16 V158 variant, CD8α hinge, and transmembrane domains, along with signaling domains from CD3ζ and CD28 or 4-1BB signaling domains [191,223]. Two completed first-in-human pilot studies already showed the efficacy (safety and antitumor activity) of infusing autologous T-cells engineered to express CD16V-41BB-CD3 ζ, in combination with rituximab, in patients with CLL and B-NHL (NCT02315118), and R/R B-NHL (NCT02776813) [325].

#### 3.6.3. CD30

This antigen belongs to TNF receptor superfamily, and CD30 signaling is involved in regulation of cytokine secretion, cell proliferation and survival [326]. CD30 is highly expressed in most B-NHL [326,327,328]. Brentuximab vedotin, an ADC comprising a human anti-CD30 chimeric Ab covalently bound to the microtubule-disrupting agent monomethyl auristatin E, was approved in R/R B-NHL after autologous stem cell transplantation and anaplastic large-cell lymphoma [326]. One phase II trial is now evaluating brentuximab vedotin in combination with three chemotherapy drugs (doxorubicin, vinblastine, and dacarbazine) for B-NHL (Table 4). Anti-CD30 CAR-T cells therapies have demonstrated significant clinical responses in early clinical trials of patients with B-NHL (Table 4) [290,291]. There are other ongoing clinical trials with different CD30 CAR-T cell constructs in R/R lymphomas addressing ways to improve outcome (Table 4) (reviewed in [329,330]).

#### 3.6.4. CD37

CD37 is expressed almost exclusively on hematopoietic cells with high expression on mature B-cells, including their malignant counterparts [331]. A large number of CD37 targeting agents have been developed including mAbs, ARCs, ADCs, and CAR-T cells [332,333,334,335]. Among them, the anti-CD37 BI856826, initially reported as a valid therapeutic target in B-NHL (NCT01403948) [336] and CLL (NCT02759016) [334], did not undergo further clinical development upon the decision of sponsors to discontinue the trials. A phase I/II study is evaluating the safety and efficacy of the ARC ^177^Lutetium-lilotomab satetraxetan (betalutin) for treatment of relapsed B-NHL (Table 4) [289]. In contrast to other newly developed CD37 targeting agents, the duoHexaBody-CD37 (GEN3009, a novel biparatopic CD37-bispecific IgG1 with an E430G hexamerization- enhancing single point mutation in the Fc-domain), mediates potent CDC in addition to ADCC and cellular phagocytosis [131]. A first-in-human clinical trial is evaluating GEN3009 in patients with R/R B-NHL (Table 4). Naratuximab emtansine (IMGN529, Debio1562) is an ADC anti-CD37 mAb conjugated to maytansine-derived microtubule disruptor; it was investigated alone in CLL and R/R B-NHL (NCT01534715) [337], and in combination with rituximab in R/R DLBCL, FL and MCL (NCT02564744), where the treatment was well-tolerated and resulted in complete response rates and a manageable safety profile [338]. The second ADC, AGS67E, is an anti-CD37 mAb conjugated to monomethyl auristatin E, that showed a favorable safety profile in a phase I clinical trial in patients with CLL and R/R B-NHL (NCT02175433) [339]. Extended studies are awaited.

#### 3.6.5. CD46

CD46 (also referred to as membrane cofactor protein, MCP) is coexpressed as four predominant isoforms on almost all cell types, and is overexpressed on a variety of human tumor cells [340]. Clinical and experimental data support an association between increased CD46 expression, malignant transformation, and metastasizing potential [340]. More recently, CD46 has been reported as a driver of metabolic processes [340]. The ADC anti-CD46-monomethyl auristatin E (FOR46) demonstrated an acceptable toxicity profile in a phase I study to treat patients with R/R MM (NCT03650491) [341].

#### 3.6.6. CD47

This ubiquitous antigen belongs to the immunoglobulin superfamily [342], and exhibits various cellular functions with multiple binding partners, such as inhibition of phagocytosis through an interaction with signal-regulating protein alpha (SIRPα) on the surface of phagocytic cells [343]. Many clinical trials have started to evaluate numerous anti-CD47 mAbs (and derivatives) in the treatment of solid tumors and hematological neoplasms including B cell diseases [343,344]. Monotherapy phase I clinical trials of anti-CD47 Abs often yielded limited signs of efficacy compared with combination strategies [343]. Among them, the humanized anti-CD47 mAb magrolimab selectively binds to CD47 expressed on tumor cells and blocks the interaction of CD47 with SIRPα, thus allowing the activation of macrophages, resulting in specific tumor cell phagocytosis [345]. Furthermore, blocking CD47 signaling activates an antitumor T-lymphocyte immune response and T-mediated cell killing [345]. Of interest, the phase II study (NCT04778397) enrolling patients with AML or myelodysplastic syndrome who received magrolimab in combination with azacytidine, was paused in 2022 following suspicions of safety issues [346]. Thus, particular attention should be paid to magrolimab, which is currently tested in combination with standard therapies in several trials in B-NHL (Table 4). A second anti-CD47 (JMT601/CPO107) is a bispecific fusion protein in which the CD47 binding fragment SIRPα is covalently fused to ofatumumab (an anti-CD20); it thus targets both CD20 and CD47 on tumor cells and leads to ADCC and CDC [344]. A phase I/II multicenter clinical trial in CD20^+^ B-NHL patients is starting (Table 4).

#### 3.6.7. CD56

This antigen, primarily expressed in cells of neuroendocrine origin as well as in NKs and some T cell subtypes, is overexpressed in several solid and hematologic malignancies, including MM [347]. The ADC lorvotuzumab mertansine (IMGN901) combines the anti-CD56 Ab lorvotuzumab covalently bound to the cytotoxic maytansinoid effector. A phase I trial of lorvotuzumab mertansine provided evidence of safety and signals of clinical activity for this agent in R/R MM (NCT00346255) [348], warranting its further clinical development as part of combination regimens for the management of MM.

#### 3.6.8. CD74

Otherwise known as HLA-DR-associated invariant chain, CD74 functions as an MHC class II chaperone and as a high affinity receptor for the pro-inflammatory cytokine macrophage migration inhibitory factor [349]. CD74 is overexpressed in some B-NHL [349]. A phase I study with the anti-CD74 mAb milatuzumab [350] showed no objective responses in R/R MM (NCT00421525) [351]. ASTRO-001 is a fully human ADC anti-CD74 incorporating maytansinoid [352]; a phase I study of ASTRO-001 is recruiting patients with advanced B cell malignancies including DLBCL, FL and MM (Table 4) [294].

#### 3.6.9. CD79B

CD79 is the BCR’s signaling component (Figure 1b) and is composed of a heterodimer of CD79A and CD79B, both of which induce internalization of the BCR [353]. CD79 is strongly expressed in most types of B-NHL and CLL but weakly expressed on normal cells (with restriction to pre-B and mature B cells) [353]. Polatuzumab vedotin is an ADC humanized anti-CD79B Ab conjugated to monomethyl auristatin E, that showed significant clinical activity in phase I trials to treat patients with FL and DLBCL [353]. In 2019, it was FDA-approved in combination with bendamustine and rituximab for patients with R/R DLBCL after at least two prior therapies [353]. Two phase I trials are evaluating polatuzumab vedotin in combination with several conventional chemotherapies for untreated or R/R patients with DLBCL and other B-NHL (Table 4).

#### 3.6.10. CD156/a Disintegrin and Metalloprotease 17 (ADAM17) (Also Referred to as TNF-α Converting Enzyme/TACE)

The cell surface protease ADAM17 is overexpressed in certains solid and hematological tumors [354], and the development of a large variety of inhibitors against the enzymatic activity of ADAM17 has made it into clinical evaluation in cancers [320]. Among these ADAM17 inhibitors, aderbasib (INCB7839) [355] entered a phase I/II clinical trial, to be used along with rituximab as consolidation therapy after an autologous hematopoietic cell transplant for patients with DLBCL (NCT02141451) (Table 4). The short-term results suggest applicability as relapse preventing therapy for DLBCL [356].

#### 3.6.11. CD307/Fc Receptor-like 5 (FcRL5)

CD307/FcRL5, specifically expressed within the B cell lineage [357], belongs to transmembrane FcRL family proteins with sequence homology to classical Fc receptors, which can promote B cell proliferation and activation [317]. Present on malignant cells from patients with MM, CLL and MCL, CD307 may be therefore a potential therapeutic target for the treatment of these diseases [358]. Cevostomab (BFCR4350A) is a BiTE anti-FcRL5/CD3 Ab being evaluated in an ongoing phase I, dose escalation and expansion trial (Table 4) [295].

#### 3.6.12. G-Protein Coupled Receptor Family C Group 5 Member D (GPRC5D)

GPRC5D is ubiquitously expressed on healthy and malignant plasma cells, and high expression on MM cells associated with adverse prognosis [359]. Talquetamab is a BiTE duobody anti-GPRC5D/CD3 currently evaluated in a phase I study, in which it is administered both intravenously or subcutaneously in patients with R/R MM (Table 4); it already shows a high clinical response rate and appears well-tolerated, allowing the next exploration of dosing strategies [296].

## 4. Targeting Metabolic Reprogramming in B Cell Malignancies—Preclinical and Clinical Studies

### 4.1. Metabolic Reprogramming in B Cell Malignancies

#### 4.1.1. The Metabolism in Cancer Cells, the Warburg Theory

The main characteristics of tumor cells (e.g., evading cell death, insensitivity to anti-growth signals, limitless replicative potential, tissue invasion and metastasis, sustained angiogenesis) were defined two decades ago as “the hallmarks of cancer” [360]. Ten years later, the same authors updated this model of acquired capabilities of cancer by including two extra features that emerged as crucial factors for cancer growth and progression, e.g., metabolic reprogramming and immune evasion [361]. When considering the current treatments of B cell malignancies, both hallmarks are very promising and intensively studied sources of inspiration for the development of new therapeutic strategies. Besides the excitement for immunotherapy with a tremendous number of engineered CAR-T/CAR-NK cells strategies (Section 3.4), the discovery of metabolic vulnerabilities in tumor cells is also likely to profoundly impact the development of new therapeutic options [362].

**Figure 3:**
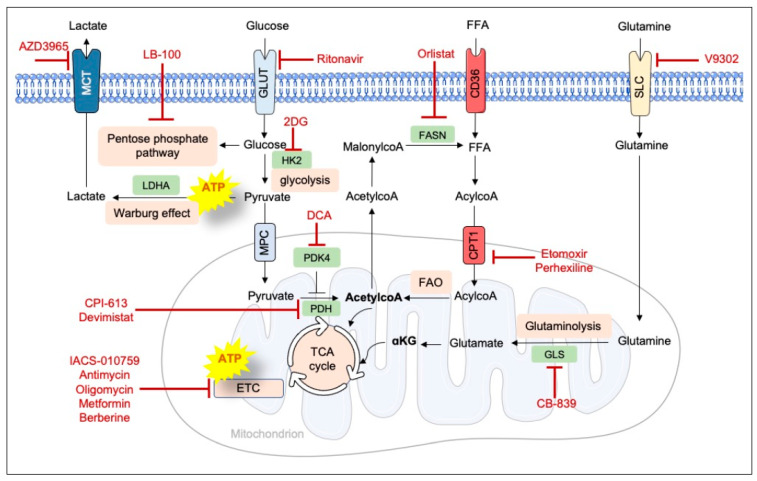
The cell’s main metabolic pathways and the corresponding inhibitors. Glucose, free fatty acids (FFAs) and glutamine enter cells via glucose transporters (GLUT), CD36, and solute carrier (SLC) transporters respectively. Once inside the cells, these substrates are catabolized by various metabolic reactions in the cytosol and the mitochondria in the tricarboxylic acid cycle (TCA). Cofactors produced by the TCA are used by the electron transport chain (ETC) to produce ATP. Some cancer cells shift their metabolism (and specifically glycolysis) towards lactate production; ATP is generated outside the mitochondria. Lactate can then be exported from the cells via monocarboxylate transporters (MCTs). Inhibitors are shown in red, key enzymes are labeled in green, and metabolic pathways are shown in pink. αKG, α-ketoglutaric acid; FASN, fatty acid synthase; FAO, fatty acid oxidation; GLS, glutaminase; HK2, hexokinase 2; LDHA, lactate dehydrogenase A; PDH, pyruvate dehydrogenase; PDK4, pyruvate dehydrogenase kinase 4.

To compensate for its elevated bioenergetic needs associated to increased proliferation and biosynthesis, tumor cells must adapt and reprogram their metabolism to increase nutrients import. In particular, a dramatic increase in glucose uptake and a shift toward its oxidation through aerobic glycolysis (referred to as the “Warburg effect”) is observed in most tumor cells (Figure 3) [362]. Glucose is first converted into pyruvate through 10 enzymatic reactions, the first of which is catalyzed by hexokinase 2 (HK2) (Figure 3). Pyruvate, the final product of glycolysis, is further transformed into lactate by the lactate dehydrogenase-A (LDHA) (Figure 3). Another major alternative to produce energetic ATP occurs through mitochondria, where the oxidation of various nutrients (including amino acids such as glutamine, or lipids such as free fatty acids/FFA) can feed the tricarboxylic (TCA) cycle (Figure 3). The TCA can then activate mitochondrial respiration through the electron transport chain, which promotes oxidative phosphorylation (OXPHOS) and silences the Warburg effect and the shift to glycolysis (Figure 3).

#### 4.1.2. Specific Metabolic Features of B Cell Malignancies

While the Warburg effect describes the typical metabolic response in aggressive B-NHL [363], other B cell malignancies favor mitochondrial respiration, such as CLL.

*Glycolysis:* the expression of the rate limiting enzyme (HK2, an indicator of glycolytic activity) is often upregulated in B-NHL [364,365]. No change in glycolysis is observed in CLL, although an increase in glycolytic capacity and reserve appears linked to drug resistance [366]. As a consequence, the blockade of the GLUT4 glucose transporter by the HIV protease inhibitor ritonavir leads to in vitro cell death in CLL [367] and potentiates the efficacy of metformin in MM cell lines and xenograft models [368]. A recent clinical trial demonstrated the safety, tolerability and feasibility of metformin + ritonavir combination therapy in patients with R/R MM or CLL (NCT02948283) [369]. Furthermore, the expression of lactate dehydrogenase A (LDHA) is upregulated in NHL [370] including MM [210]. Because of the involvement of glycolysis in B-NHL progression, the targeting of lactate trafficking through the blockade of monocarboxylate transporter 1 (MCT-1) by AZD3965 shows very promising results in NHL [371], including DLBCL [372] A phase I clinical trial of AZD3965 combined to rituximab in DLBCL has recently been completed (NCT01791595) [373]. Independently of glycolysis, one regulatory mechanism has been described in B-cell malignancies by which glucose is redirected toward glycolysis or the pentose phosphate pathway (PPP), offering new options with the LB-100 inhibitor [374].

*Mitochondrial OxPhos:* an increase in OxPhos has been observed in MCL [375], MM [376] and CLL [377,378]. The increase in OXPHOS in CLL leads to increase in the oxidative stress [379], and forcing mitochondrial respiration through PI3K/AKT hyperactivation leads to toxic accumulation of ROS, resulting in CLL cell death [380]. In DLBCL, the combination of AZD3965 with the mitochondrial respiration inhibitor IACS-010759 in B cell lines and xenografts suggests a clear clinical advantage compared with monotherapy [381]. The novel mitochondrial disrupter CPI-613 (devimistat, a lipoate analog) inhibits the mitochondrial enzymes pyruvate dehydrogenase and α-ketoglutarate dehydrogenase in the TCA cycle [382,383]. A phase II study with CPI-613 is now recruiting patients with R/R NHL, including those with high-risk translocations of Myc, BCL2 and/or BCL6 (NCT03793140).

*Lipids:* beside carbohydrates metabolism, the metabolism of lipids is also affected in B-cell malignancies [264]. Lipid metabolism is upregulated in BL [384]. In CLL, high expression of lipoprotein lipase LPL is associated with a shorter treatment-free survival and a trend of a shorter median overall survival [385]. Its overexpression causes an increase in the β-oxidation of FFAs [386], while its inhibition by orlistat induces apoptosis of CLL cells [387]. Orlistat also induces cell death in MCL by inhibiting fatty acid synthase (FASN) [388] while pharmacological or genetic inhibition of FASN in DLBCL also induces apoptosis [389]. The antigen CD36 that facilitates FFA uptake by cells, is upregulated in CLL, and its inhibition by anti-CD36 neutralizing Abs or sulfosuccinimidyl oleate (SSO) leads to apoptosis [390]. Similarly, the blockade of FFAs import into mitochondria via their β-oxidation by perhexiline (inhibitor of carnitine palmitoyltransferases (CPT), essential for transporting long chain fatty acid into mitochondria) [391] or the irreversible inhibitor of CPT-1, etomoxir [377], induces CLL cell death.

*Amino acids:* amino acids play a crucial role in the progression of B-cell malignancies. In peculiar, addiction of tumor cells to glutamine is observed in many tumors including MM [392] and CLL with 11q deletion [393], and in the Burkitt lymphoma cell line P493 [394]. The glutaminase inhibitor CB-839 (telaglenastat) has been tested in phase I clinical trial in MM and WM, but the results are not yet available (NCT02071888).

#### 4.1.3. Involvement of the Tumor Microenvironment in the Adaptative Metabolism

Often, tumor cells depend on microenvironmental non-malignant cells for survival. Indeed, while some intrinsic common metabolic characteristics can be observed in B cell malignancies, metabolism can be modified by the contact of tumor cells with the adjacent tumor microenvironment (TME) [395,396]. For instance, interaction of tumor CLL cells with surrounding bone marrow (BM) stromal cells is shown to induce a shift toward glycolysis [379,397], that is in part mediated by Notch/c-Myc signaling [379]. Protection by the TME involves the transcriptional regulation of genes involved in redox homeostasis [398]. Furthermore, the protection of CLL cells from oxidative stress is also handled through a metabolic collaboration with stromal cells which transform cystine to cysteine, and cysteine transport to CLL cells allows the synthesis of GSH to promote cell survival and drug resistance [399]. As recently reviewed by Nie et al. [378], the agents targeting metabolic enzymes or signal pathways activated in CLL by lymph nodes (LN) microenvironment (converging to glucose, purine and glutamine metabolism) may effectively impede the progression of CLL [400]; these data provide further insight into the crucial metabolic changes driven by the CLL LN microenvironment and the prominent use of amino acids as fuel for the TCA cycle, in view of new therapeutic vulnerabilities. A high mitochondrial respiration in MM and B-ALL cells appears to be the consequence of the direct transfer of mitochondria from stromal to tumor cells [401,402]; this phenomenon in MM cells is driven by CD38 [402]. Tumor-associated adipocytes support MM cells through various mechanisms, including metabolic reprogramming of MM cells which involves MM cell–induced lipolysis in BM adipocytes, upregulation of FA transporters 1 and 4 on MM cells which then mediate the uptake of secreted free FAs, leading to enhanced MM proliferation [403].

In contrast, tumor-associated stromal cells can metabolically stimulate the growth and metastasis of cancer by producing a “reverse Warburg effect” [404,405]. Thus, stromal cells shift from glycolysis to mitochondrial respiration in cases of B-NHL [406,407]; in particular, the lactate trafficking that promotes tumor growth in NHL is caused by the upregulation of MCT4 in tumor-associated macrophages and the concomitant upregulation of MCT-1 in tumor cells [371,408]. The blockade of lactate by AZD3965 leads to NHL cell apoptosis [371,408].

In conclusion, these examples of metabolic vulnerabilities open up new opportunities for the treatment of B cell malignancies by cutting off the tumor’s supplies of energy substrates.

### 4.2. Targeting Metabolism in B Cell Malignancies: Clinical Perspectives

#### 4.2.1. Metabolic Features as Biomarkers of B Cell Malignancies

Metabolomic approaches on plasma of patients with B cell malignancies may allow to identify metabolic biomarkers for early diagnosis and prediction, prognosis and treatment [409]. Although many metabolic biomarkers have already shown their clinical validity, many others have not yet been subjected to extensive testing to demonstrate their clinical usefulness in B-cell malignancies. For instance, three metabolic subtypes have been identified in nine B-NHL model cell lines according to their preferential use of glucose, glutamine or both as a source of energy [410]. As an attempt to perform a classification of B-NHL according to their specific metabolic profile, a pilot study was performed on the plasma samples of 66 NHL patients, compared with 96 matching controls [411]. A significant difference was observed between the metabolites produced by DLBCL, CLL, MM, NHL patients’ plasma (but not FL) and their respective control population, allowing to discriminate these patients and healthy controls [411]. Although the NHL cohort size was rather small, the results already suggest a classification of NHL subtypes based on plasma metabolites [411]. In MM, the serum metabolic profiling shows a specific signature after complete remission [412]. A similar analysis of serum from 47 childhood ALL patients lead to the identification of 30 potential biomarkers (17 detected in positive mode and 13 in negative mode) [413]; in particular, deregulated glycerophospholipid metabolism in ALL patients appeared to represent an underlying metabolic pathway associated with disease progression [413]; this non-invasive diagnosis approach may especially be relevant as diagnosing ALL requires BM puncture procedure.

In CLL, IGHV mutational status correlates with disease-related metabolic profiles [366,414]. Several key glycolytic genes (*PFKP, PGAM1 and PGK1*) were found to be down-regulated in samples harboring mutated immunoglobulin variable heavy-chain. Furthermore, 8q24 copy number gains, 8p12 deletions, 13q14 deletions and ATM mutations were identified as determinants of cellular respiration [366]. Such heterogeneity in the energy metabolism of CLL cells could be therapeutically exploited in the selection of therapeutic strategies. In DLBCL, a genome-wide array study identified three clusters including one defined by increased OXPHOS and expression of genes involved in mitochondrial oxidative phosphorylation (OxPhos-DLBCL). OxPhos-DLBCL also overexpresses proteasome and apoptosis regulators, suggesting a better response to drugs targeting mitochondrial function, proteasome and BCL2 family [415]. A further characterization of these three DLBCL subtypes detailed the metabolic signature of each of the subtypes [415]. The blockade of FAO and GSH synthesis is selectively toxic to OxPhos-DLBCL [416]. Conversely, the absence of functional BCR signaling in OxPhos-DLBCL renders it insensitive to BCR inhibition contrary to non-OxPhos-DLBCL [417], resulting in enhanced glycolysis [416]. More recently, a very comprehensive study identified the glycolytic enzyme glyceraldehyde-3-phosphate dehydrogenase (GAPDH) as a biomarker for distinguishing between OxPhos-DLBCL (GAPDH^low^) and non-OxPhos-DLBCL (GAPDH^high^) and a clinical marker for predicting overall survival in patients treated with R-CHOP [418].

#### 4.2.2. New Strategies for Targeting Metabolism in B Cell Malignancies and Optimizing Current Treatments

While metabolic inhibitors have been used for decades for the treatment of metabolic disorders, the acknowledgement of metabolic reprogramming as a crucial feature of cancer biology has recently started to open new perspective for cancer treatment, with the repositioning of commercial metabolic drugs, or the design of new inhibitors evaluated in clinical trials [419,420]. As the inhibition of major metabolic pathways may have a dramatic impact on both tumor and normal cells, current experience shows that the best potential of metabolic inhibitors to treat tumor, comes from their combination with classically used drugs, as a way to overcome drug resistance [421]. Importantly, while tumor progression correlates to a change in metabolic profile, acquisition of resistance to drug is, most of the time, also linked to a change in bioenergetics. Thus, resistance to chemo-immunotherapy in CLL correlates to the activities of glycolytic enzymes LDHA and pyruvate kinase M2 (PKM2), leading to the development of a metabolic score as a diagnostic tool in CLL [422]. A larger CLL screening study revealed that the metabolic state of leukemic cells is associated with drug sensitivity; in particular, higher glycolytic activity is linked to increased resistance towards drugs (including venetoclax, ibrutinib, R-CHOP, rituximab, proteasome inhibitors) thus shedding some light on metabolic vulnerabilities displayed by resistant cells [366].

*Venetoclax:* two main metabolic patterns emerge in venetoclax resistant B-NHL and CLL. First, mitochondrial OxPhos is increased in response to venetoclax in B-NHL, rendering them more sensitive to respiration inhibitors such as antimycin, oligomycin, IACS-010759, metformin or berberine [423,424,425,426]. Second, inhibition of LN-derived glutamine import with V9302 attenuates LN-induced resistance of CLL cells to venetoclax [400]; a higher glycolytic activity is also part of the resistant phenotype [366].

*Ibrutinib:* ibrutinib treatment which blocks BCR signaling also interferes with metabolic pathways in CLL, decreasing mitochondrial oxidative respiration, glycolysis and glutamine metabolism [400,427,428] An increase in glutamine uptake is also observed in ibrutinib-resistant CLL cells, and etomoxir treatment (that blocks the mitochondrial import of FAs and their βeta-oxidation), has a cytotoxic effect on ibrutinib resistant CLL cells [377]. In contrast, ibrutinib-resistant MCL cells display increased OxPhos, and combining ibrutinib to IACS-010759 blocks tumor growth [375]. Ibrutinib resistance also correlates to higher glutaminolysis, that renders glutaminase inhibitor CB-839 efficient at killing ibrutinib-resistant MCL cells [429].

*R-CHOP:* Low GAPDH expression level correlates to DLBCL patients’ resistance to R-CHOP treatment [418,430], and their sensitivity to metabolic inhibitors targeting mammalian target of rapamycin complex 1 (mTORC1) signaling, OxPhos and glutaminolysis [418]. In a preliminary clinical trial, four DLBCL patients with GAPDH^low^ tumors received a combination therapy involving kidrolase (asparaginase), metformin, and temsirolimus (mTOR inhibitor), tumor regression was reported in three out of four patients for the first two weeks of treatment, but relapsed after longer treatment, with a median response duration of six months [430]. This preliminary study suggests that combination of metabolic inhibitors may offer a treatment strategy for metabolically active cancers [430].

*Rituximab:* acquisition of resistance to rituximab appears related to an increase in glycolysis in BL and DLBCL cell lines, and downregulation of HK2 by 2-Deoxy-D-glucose (2DG) glucose competitive inhibitor, metformin, idelalisib or temsirolimus overcomes resistance and enhances the cytotoxic effect of rituximab [431]. Similarly, the blockade of lactate transporter MCT1 by AZD3965 combined to rituximab leads to tumor regression in a BL mouse xenograft [432] as well as in patients with DLBCL (phase I NCT01791595, completed in 2020) [373]. An enhancement of rituximab response is observed in DLBCL when combined with metformin [433] or pyruvate dehydrogenase kinase 4 (PDK4) inhibitor dichloroacetate (DCA) [434]. Both temsirolimus and everolimus are already FDA-approved for the combined treatment with rituximab of R/R MCL, DLBCL and FL [435].

*Proteasome inhibitors:* in MM cell lines and a MM mouse xenograft, the response to proteasome inhibitors is increased when combined to the glutaminase inhibitor CB-839 [436] or to DCA [437].

#### 4.2.3. Metabolism as a Tool to Boost Immunotherapy

In the last years, the development of immunotherapy has been quite impressive for the treatment of various tumors including B-cell malignancies. Despite some very promising results, this new therapy is facing long term efficacy issues, due to the difficulty to maintain the proliferation of immune cells in an immunosuppressive TME. One major limitation is the availability of nutriments in the TME, for which immune and tumor cells compete; the high avidity of tumor cells for energy substrates, especially glucose, often results in exhaustion of immune cells, ending with tumor immune evasion [438]. Moreover, the accumulation of immune suppressive metabolites in the TME has negative effects on the immune system, in particular lactate production by tumor cells can inhibit the activity of B and T cells [439]. Complementing immunotherapy with molecules that modulate metabolism therefore emerges as a solution of choice to optimize immunotherapies, including chimeric antigen receptor (CAR) T cells [440,441]. For instance, glutaminolysis in DLBCL interferes with immunotherapy by regulating positively PD-L1 expression [442]. Two phase I clinical trials in B-NHL evaluated the combinations of pembrolizumab with troriluzole (BHV-4157), an inhibitor of glutamate (NCT03229278, completed in 2020), or ciforadenant, an antagonist of the adenosine A2A receptor (NCT03454451, 2018–2025). These innovative approaches deserve to be pursued.

## 5. Concluding Remarks and Perspectives

Today, a key challenge in using FDA-approved drugs in the current treatment of B cell malignancies (Figure 2a) is that these therapies must simultaneously eliminate tumor cells and preserve/reestablish normal hematopoiesis. However, the effectiveness of these anticancer therapies is sometimes limited by primary and acquired resistance [1]. Drug-mediated T cell exhaustion may lead to the possibility of immune escape of hematological malignancies [1]. Residual disease and/or resistance in patients treated with Bcl-2/BTK/PI3K inhibitors (venetoclax, ibritunib, idelalisib, etc.) is associated with genomic resistance (mutations, activation, instability, p53 aberrations.) as well as non-genomic, acquired resistance through (re)activation of signaling survival pathways (PI3K, NF-κB, MYC, etc.), cancer stem cells, and the tumor microenvironment [60,443,444,445,446,447]. The main adverse effects of mAbs therapies include cytokine release syndrome, neurotoxicity, and on-target/off-tumor toxicity resulting from a direct attack on normal tissues that have shared expression of the target antigen [223,448]. To improve clinical outcomes (residual and progressive disease) and immune function in patients with B cell malignancies, while evading the emergence of drug resistance, newer drugs that inhibit the same targets as the current treatments are in continuous clinical development. For instance, ongoing trials focus on overcoming venetoclax resistance by targeting the BCL2 family members Mcl-1 and Bcl-x_L_, and evaluate more highly selective BTK and PI3Kδ inhibitors with fewer off-target effects. These second-generation drugs, although more potent, are not likely to be used only as single agents. Drug combinations can favor compounds with synergistic antitumor effects and ensure manageable toxicity. Thus, the combinations of BTK inhibitors with BCL2 inhibitors and/or rituximab (anti-CD20 mAb), or PI3K inhibitors with BCL2 inhibitors, are of interest.

Importantly, this review outlines the increasing importance of therapeutic mAbs (mono-/bi-specific, ADC, BiTE, CAR-T cells, Bi-CAR-T cells, CAR-NK cells) as the predominant treatment modality for B cell malignancies. To face the resistance mechanism, BiTEs and CAR-T cells redirect T cells to target antigen-expressing tumors; novel BiTEs and CAR-T cells with various costimulatory signals and delivery systems, as well as Bi-CAR-T cells (anti-CD19/CD20 CAR-T, anti-CD19/CD22 CAR-T, and anti-CD20/CD22 CAR-T) may therefore represent an effective solution to the challenge of antigen escape in immunotherapy (Table 3). As already planned for the treatment of solid tumors [449], BiTEs and CAR-T cells could be combined with standard chemotherapy and/or targeted therapies of B cell malignancies to reduce the tumor burden and/or modulate the immune response. Recently, CAR-T cells and BiTEs have been engineered into a single immunotherapy platform (BiTE-secreting CAR-T) for T-cell-directed therapy of solid tumors [450,451]. BiTEs secreted by CAR-T cells exhibit potent antitumor activity in vitro and in vivo with significant sensitivity and specificity, demonstrating a promising strategy in solid tumor therapy [450,451]. This approach could have therapeutic application in hematological malignancies. Lastly, compared with CAR-T cells, CAR-NK cell constructs offer advantages, including better safety, and multiple mechanisms for activating cytotoxic activity [452,453,454]. For therapy of B cell malignancies, equipping anti-CD19 CAR-NK cells with on-board cytokines or chemokines might improve clinical efficacy by enhancing both persistence and cytotoxicity against tumor cells.

The consideration of other TAAs (including BCMA, BAFF-R, ROR1, CXCR4, CS1, FcRL5, GPRC5D, etc.) as druggable targets for B cell malignancy immunotherapy, has promoted the development of novel inhibitors, with some of them under single or combined evaluation in clinical trials for a variety of patients with B cell malignancies (Table 4). Most TAAs show their potential for ADC, BiTE, CAR-T, and CAR-NK therapies. Regarding the PD1/PD-L1 axis responsible in part for cancer immune escape, immune checkpoint blockade therapies with anti-PD1/anti-PD-L1 mAbs in combination with BTK (ibrutinib), anti-CD38 (isatuximab) or anti-CD19 CAR-T (axicabtagene ciloleucel) may optimize the landscape of B-NHL therapy.

Targeting additional intracellular signaling pathways contributing to B malignant cell dysfunction may also prove efficacious. For example, ruxolitinib, a potent JAK1/2 inhibitor, demonstrates antitumor activity as a single agent in MM (NCT03110822) [455]. Anvatirsen, an antisense oligonucleotide inhibiting STAT3, combined with durvalumab + tremelimumab is well-tolerated in patients with R/R DLBCL (NCT02807454) [311]. The efficacy and safety of a combination of panobinostat (a pan-histone deacetylase inhibitor) with bortezomib + dexamethasone is being evaluated in MM patients having received two or more lines of treatment (NCT02654990). Tazemetostat/EPZ-6348, which inhibits the histone methyl transferase EZH2, shows safety and antitumor activity in patients with DLBCL (NCT02889523) and FL (phase I/II NCT01897571) [456,457,458]. Combination therapy with temsirolimus, an inhibitor of the oncogenic kinase mTOR, and lenalidomide demonstrates encouraging activity in patients with DLBCL and FL (NCT01076543) [459]. Ponatinib is a third-generation tyrosine kinase inhibitor with a wide spectrum of kinase inhibition [460]; it targets BCR-ABL1, an abnormal tyrosine kinase that is expressed in chronic myeloid leukemia and Philadelphia chromosome-positive (Ph^+^) ALL; ponatinib shows clinical activity in R/R Ph^+^ (BCR-ABL) ALL [461], and in the first-line setting in combination with standard chemotherapy (NCT01641107, [462]; NCT02776605, [463]) or blinatumomab (NCT03263572) [464]. A Phase III study is comparing the efficacy and the safety of the first in-class selective inhibitor of nuclear export, selinexor, in combination with bortezomib + dexamethasone vs. bortezomib + dexamethasone in patients with R/R MM (NCT03110562) [465]. Pharmacological inhibition of the bromodomain and extra-terminal (BRD/BET) family proteins block downstream components of BCR signaling, downregulate Bcl-2 transcription, and suppress NF-κB signaling in CLL and B-NHL [466]; among novel single-molecules cotargeting BRD4 and other tumor targets recently developed, SRX3177 targeting CDK4/6-PI3K-BRD4 and SRX3305 targeting BTK-PI3K-BRD4, demonstrate preclinical activity against MCL [467], CLL [468], and MCL [469]. These studies underscore the potential effectiveness of these novel multi-action small molecule inhibitors, alone or combined, for potential treatment of B tumors.

Last, but not least, metabolic changes in tumor cells represent a novel opportunity for combination therapy approaches [470]. Metabolic reprogramming is linked to oncogenic transformation. One hallmark of BCL2, PI3K, BTK, and mTOR proteins and some TAAs such as CD38, concerns their participation in tumor metabolism. Activation of these oncogenic pathways makes tumors more metabolically active, and conversely, active metabolism upregulates BCL2, PI3K, BTK, and mTOR proteins, inhibiting susceptibility to cell death. In a consistent way, a few clinical trials have started to include metabolic inhibitors (targeting for instance glycolysis, OxPhos, amino acids) with venetoclax, ibrutinib and other drugs, to overcome the limitations of targeted therapeutic strategies in lymphoid malignancies. As metabolic reprogramming is closely linked to tumor B cells’ microenvironment and to immunoevasion, strategies targeting these crosstalks may also open new avenues for overcoming therapeutic resistance.

To conclude, most therapeutic drugs have underscored our advancement in the understanding of the biology of malignant B cells, and has improved outcomes for many patients. In the search for better efficacy and safety, the status of therapies in B cell malignancies is continually advancing with emerging concepts in therapy and evolving results from clinical protocols. A large variety of more effective and selective inhibitors and targeted combinations are being evaluated in these diseases, and it is expected that some of these new compounds will proceed into the clinic in the near future.

## Figures and Tables

**Table 4:** Selected ongoing and now-recruiting clinical trials of novel anti-TAA inhibitors in B cell malignancies.

Drug	Class	Disease Setting	Phase	Study	References
Belantamab mafodotin/ GSK2857916	ADC anti-BCMA-monomethyl auristatin F	R/R MM	I/II I/II	NCT03848845 Active, 2018- Alone or combined with pembrolizumab NCT04126200 NCT03544281 NCT03715478 Recruiting, 2018- Alone or combined with current therapies	[273]
Teclistamab/ JNJ-64007957	BiTE anti-BCMA/CD3	R/R MM	I/II I III	NCT04557098 Active, 2017–2024 NCT04586426 Recruiting, 2019–2024 Combined with talquetamab or daratumuab or pomalidomide NCT05083169 Recruiting, 2021–2026 daratumumab compared with current therapies	[278]
Elranatamab/ PF-06863135	BiTE anti-BCMA/CD3	Refractory MM	II	NCT04649359 Active, 2020–2024	
Idecabtagene vicleucel/ bb2121	Anti-BCMA CAR-T	R/R MM	II	NCT03361748 Active, 2017–2024	[279]
BM38	Dual anti-BCMA/CD38 CAR-T	R/R MM	I	ChiCTR1900026286 Active, 2019-	[281]
BCMA-CAR-NK92	Anti-BCMA CAR-NK	R/R MM	I/II	NCT03940833 Recruiting, 2019–2022	[282]
Lanalumab/VAY-736	Anti-BAFF-R Ab	CLL	I	NCT03400176, Recruiting, 2018- Alone or combined with ibrutinib	
BAFFR-CAR T	Anti-BAFF-R CAR-T	B-ALL, R/R MCL	I	NCT04690595, NCT05370430 Recruiting, 2020–2024	
Cirmtuzumab/ UC961 newly renamed Zilovertamab	Anti-ROR1 Ab	CLL CLL, SLL, MCL R/R MCL	II I/II III	NCT04501939 Recruiting, 2020- Combined with venetoclax NCT03088878 Active, 2018–2025 Combined with ibrutinib NCT05431179 Recruiting, 2022- Combined with ibrutinib	
Pembrolizumab/MK-3475	Anti-PD1 Ab recruiting immune cells to attack tumor B cells	R/R CLL, B-NHL	II	NCT02332980 Active, 2015–2023 Alone or combined with ibrutinib or idelalisib	[283]
		High-Risk or R/R CLL/SLL	II	NCT03204188 Active, 2017–2030 Combined with ibrutinib, fludarabine	
		R/R WM	II	NCT03630042 Recruiting, 2018- Combined with rituximab	
		R/R B-ALL	I/II	NCT03512405 Recruiting, 2018- Combined with blinatumomab	[284]
		R/R DLBCL	I	NCT03340766 Active, 2017- Combined with blinatumomab	
Cemiplimab/ REGN2810	Anti-PD1 Ab recruiting immune cells to attack tumor B cells	B-NHL R/R MM DLBCL	I I/II I/II	NCT02651662 Active, 2016- Combined with REGN1979 (anti-CD20-CD3) NCT03194867 Active, 2016- Combined with isatuximab NCT03769181 Active, 2018- Combined with isatuximab	[285]
Atezolizumab	Anti-PD-L1 Ab	DLBCL	I/II	NCT02926833 Active, 2016–2022 Alone or combined with axicabtagene ciloleucel	[286]
Ulocuplumab/ BMS-936564	Anti-CXCR4 Ab	WM	I/II	NCT03225716 Active, 2017–2025 Alone or combined with ibrutinib	[287]
Mavorixafor/ AMD070	CXCR4 antagonist (blocks CXCL12 binding)	WM	I	NCT04274738 Active, 2020–2025 Alone or combined with ibrutinib	
CS1-CAR-T	CS1-CAR-T/IL7- CCL19	R/R MM	I	NCT03778346 Recruiting, 2018–2022	
CS1-CAR-T	CS1-CAR-T/41BB- tEGFR	R/R MM	I	NCT03710421 Recruiting, 2018–2023 following chemotherapy	
J1/Melflufen	Melphalan-p-fluoro-L-phenylalalnine (aminopeptidase substrat)	MM refractory to lenalidomide	III	NCT03151811 Active, 2017- combined with dexamethasone compared to pomalidomide + dexamethasone	[288]
Brentuximab vedotin	ADC anti-CD30-mono methyl auristatin E	R/R B-NHL	II	NCT03646123 Active, 2018–2026	
Lilotomab/ tetulomab/ betalutin	ARC anti-CD37-^177^lutetium	Relapsed B-NHL	I/II	NCT01796171 Recruiting, 2013–2026	[289]
GEN3009	DuoHexaBody-CD37	R/R B-NHL	I	NCT04358458 Recruiting, 2020–2025	
CART30	Anti-CD30 CAR-T	B-NHL	I	NCT02259556 Recruiting, 2016–2029 Combined with cyclophosphamide and fludarabine	[290]
CD30.CAR-Ts	Anti-CD30 CAR-T	B-NHL	I/II	NCT02690545 Recruiting, 2016–2038 Combined with fludarabine and bendamustine	[291]
CD30.CAR T	Anti-CD30 CAR-T	B-NHL	I	NCT02917083 Recruiting, 2018–2036	
ICAR30	Anti-CD30 CAR-T	B-NHL	I	NCT03383965 Recruiting, 2017–2025	
ATLCAR.CD30	Anti-CD30 CAR-T	B-NHL	I	NCT026663297 Active, 2016–2037	
Magrolimab/ Hu5F9-G4	Anti-CD47	R/R B-NHL	I/II	NCT02953509 Active, 2016–2026 Combined with rituximab and (in some cases) with chemotherapy	[292]
		R/R MM	II	NCT04892446 Recruiting, 2021–2024 Combined with daratumumab, or pomalidomide and dexamethasone, or bortezomib and dexamethasone	
		R/R B-NHL	II	NCT04788043 Recruiting, 2021–2027 Combined with pembrolizumab	
IBI322	Bi anti-CD47/PD-L1	ALL, CLL/SLL B-NHL	I	NCT04795128 Recruiting, 2021–2024	
TG-1801/ NI-1701	Bi anti-CD47/CD19	B-NHL B-NHL, CLL	I Ib	NCT03804996 Active, 2019–2022 Combined with ublituximab NCT04806035 Recruiting, 2021–2023 Alone or combined with ublituximab	[293]
CPO107/ JMT601	Fusion protein Anti-CD20-SIRPα	Advanced B-NHL	I	NCT04853329 Recruiting, 2021–2024	
STRO-001	ADC anti-CD74- maytansoid	DLBCL, FL, MM	I	NCT03424603 Recruiting, 2018–2023	[294]
Polatuzumab vedotin	ADC anti-CD79b-monomethyl auristatin E	DLBCL	I	NCT04231877 Recruiting, 2020–2031 Combined with rituximab, prednisone, etoposide, doxorubicin, cyclophosphamide	
		R/R DLBCL	I	NCT04739813 Recruiting, 2021–2027 Combined with venetoclax, ibrutinib, prednisone, obinutuzumab, and lenalidomide	
		R/R B-NHL	Ib/II	NCT03533283 Recruiting, 2018–2024 Combined with glofitamab	
Cevostomab/ BFCR4350A	BiTE-anti-FcRL5/CD3	R/R MM	I	NCT03275103 Recruiting, 2017–2023	[295]
Talquetamab/ JNJ-64407564	BiTE duoBody-anti-GPRC5D/CD3	R/R MM	I/II	NCT03399799 Recruiting, 2018–2025	[296]

ADAM17, a disintegrin and metalloprotease 17; ADC, antibody drug conjugate; BAFF-R, B-cell activating factor receptor; BCMA, B cell maturation antigen FcRL5, Fc receptor-like 5; GPRC5D, G-protein coupled receptor family C group 5 member D; ROR1, receptor tyrosine kinase-like orphan receptor 1; tEGFR, truncated epidermal growth factor receptor.
